# Early-life maternal care is required for the typical development of calming responses to back stroking

**DOI:** 10.1038/s42003-026-10012-6

**Published:** 2026-04-10

**Authors:** Sachine Yoshida, Akiko Harauma, Toru Moriguchi, Yousuke Tsuneoka, Kimiya Narikiyo, Kazuya Miyanishi, Makoto Kashima, Makoto Wada, Yu Hayashi, Hiromasa Funato

**Affiliations:** 1 Department of Anatomy, Faculty of Medicine, Toho University, Tokyo, Japan; 2 Department of Food and Life Science, School of Life and Environmental Sciences, Azabu University, Sagamihara, Japan; 3 International Institute for Integrative Sleep Medicine (WPI-IIIS), Tsukuba Institute for Advanced Research (TIAR), University of Tsukuba, Tsukuba, Japan; 4 Department of Biomolecular Science, Faculty of Science, Toho University, Funabashi, Japan; 5 Developmental Disorders Section, Department of Rehabilitation for Brain Functions, Research Institute of National Rehabilitation Center for Persons with Disabilities, Tokorozawa, Japan; 6 Department of Biological Sciences, Graduate School of Science, The University of Tokyo, Tokyo, Japan

**Keywords:** Somatosensory system, Physiology, Sensory processing

## Abstract

In many mammals, early interactions between caregivers and offspring involve rich physical contact during which offspring typically remain calm near the caregiver. Such contact is thought to support emotional regulation during infancy, but how prior experience shapes these mechanisms remains unclear. Here, we show that back stroking induces a calming response in human infants and mouse pups, with reduced movement. In mouse pups, back stroking further reduces heart rate, facilitates sleep onset, and attenuates stress-induced corticosterone elevations. These sleep-promoting and stress-buffering effects are absent in artificially reared pups deprived of postnatal maternal care, suggesting that early experience tunes the calming response to stroking. Transcriptomic analysis reveals reduced hypothalamic expression of the calcium channel subunit gene *Cacna1b* in artificially reared pups, and knockdown of hypothalamic *Cacna1b* in maternally reared pups abolishes stroking-induced calming. Thus, early-life maternal care and associated physical contact may shape hypothalamic circuits supporting behavioral and physiological regulation.

## Abstract (web summary)

Back stroking rapidly calms human infants and mouse pups, markedly reducing movement. This shared response requires early-life maternal care and depends on hypothalamic Cav2.2 channel activity, linking caregiving to arousal regulation.

## Introduction

Physical contact between the primary caregiver, such as the mother, and infants plays a critical role in supporting healthy emotional and physiological development. In humans, this principle is underscored by historical observations of infants raised in severely deprived conditions, such as institutionalized care with minimal caregiver interaction, who exhibited profound developmental delays and increased mortality rates^1–4^. These early findings have contributed to a widespread consensus that physical contact with caregivers is essential for optimal growth and well-being. The importance of early-life tactile input is not unique to humans. In many mammalian species, including rodents, maternal separation or early weaning leads to long-lasting behavioral and physiological changes in the offspring^5,6^. Despite the well-recognized benefits of caregiver–infant contact, the biological mechanisms through which affiliative tactile input, also referred to as affective touch, supports emotional regulation and stress buffering remain incompletely understood. Although many studies have examined the adverse effects of tactile deprivation^1,4,7^, the development of molecular mechanisms underlying the adaptive functions of tactile input, such as promoting relaxation and behavioral calmness, remains unclear.

Among the various forms of affiliative tactile input, stroking is a common and intentional behavior observed in daily caregiving practices. Parents often stroke their infant’s body to soothe distress or facilitate sleep^8^. Similar behaviors have been documented in non-human mammals. For instance, macaque infants display fewer stress-related behaviors, such as scratching and agitation, after receiving stroking from caregivers^9^. In rodents, maternal licking provides a form of tactile stimulation functionally analogous to stroking, and the amount of licking received in early life has been shown to influence stress responsiveness and emotional development in adulthood^10–13^. During stroking, low-threshold mechanosensitive C-fiber afferents and Aβ afferents are likely to be activated. In humans, C-tactile (CT) afferents, a subclass of C fibers, respond preferentially to gentle stroking especially on areas such as the back, which is typically associated with pleasant sensations^14,15^. In mice, C-low-threshold mechanoreceptors (C-LTMRs) selectively innervate hairy skin and are more densely distributed in dorsal than in ventral regions^16^. In both humans and mice, Aβ afferents are fast-conducting, myelinated low-threshold mechanoreceptors that convey innocuous tactile information, responding to dynamic skin deformation during stroking and to sustained skin indentation and pressure^17,18^. However, the molecular basis underlying the calming effects of stroking on arousal and stress during development, as well as the influence of postnatal somatosensory experience on these effects, remains unclear. To elucidate the developmental and molecular basis of this phenomenon, we examined the physiological and transcriptomic consequences of back stroking in both human infants and mouse pups, focusing specifically on the role of early-life tactile experience. We observed that back stroking reduced spontaneous movement in both species. In mice, this stimulation further reduced heart rate, facilitated sleep onset, and suppressed stress-induced hormone elevations. To evaluate the contribution of early tactile input, we compared artificially reared pups, which lacked maternal physical contact, with normally reared counterparts. We also conducted transcriptomic screening of the hypothalamus to identify genes associated with early tactile experience and performed targeted knockdown experiments to assess their role in mediating the calming effects of stroking. We provide evidence that early-life maternal care engages hypothalamic *Cacna1b*-dependent mechanisms that contribute to the development of stroking-evoked calming responses, characterized by a coordinated reduction in motor output and arousal-related physiological indices.

## Results

### Back stroking reduces spontaneous movements in human infants

To examine the real-time effects of mothers’ stroking on their infants, we first investigated how stroking affects the spontaneous movement and heart rate of their infants. Each infant was attached to the disposable adhesive electrocardiogram (ECG) electrodes on their chests, and mother-infant pairs were video-recorded during the experiment sessions. Mothers were seated on a chair with their infant on their lap and instructed to gently but firmly stroke one of three body areas: the infant’s back, the back of the head, or the lower abdomen, for 1  min each, in a randomized order. To minimize other verbal or nonverbal communication that could affect the infants’ responses, mothers were asked to refrain from making eye contact, speaking to their infant, or rocking them. Since some mothers may feel anxious about performing the procedure in an unfamiliar laboratory setting, we provided brief guidance and asked them to stroke their infant with firm contact pressure and at their usual speed, as they would normally do when soothing.

**Fig. 1:**
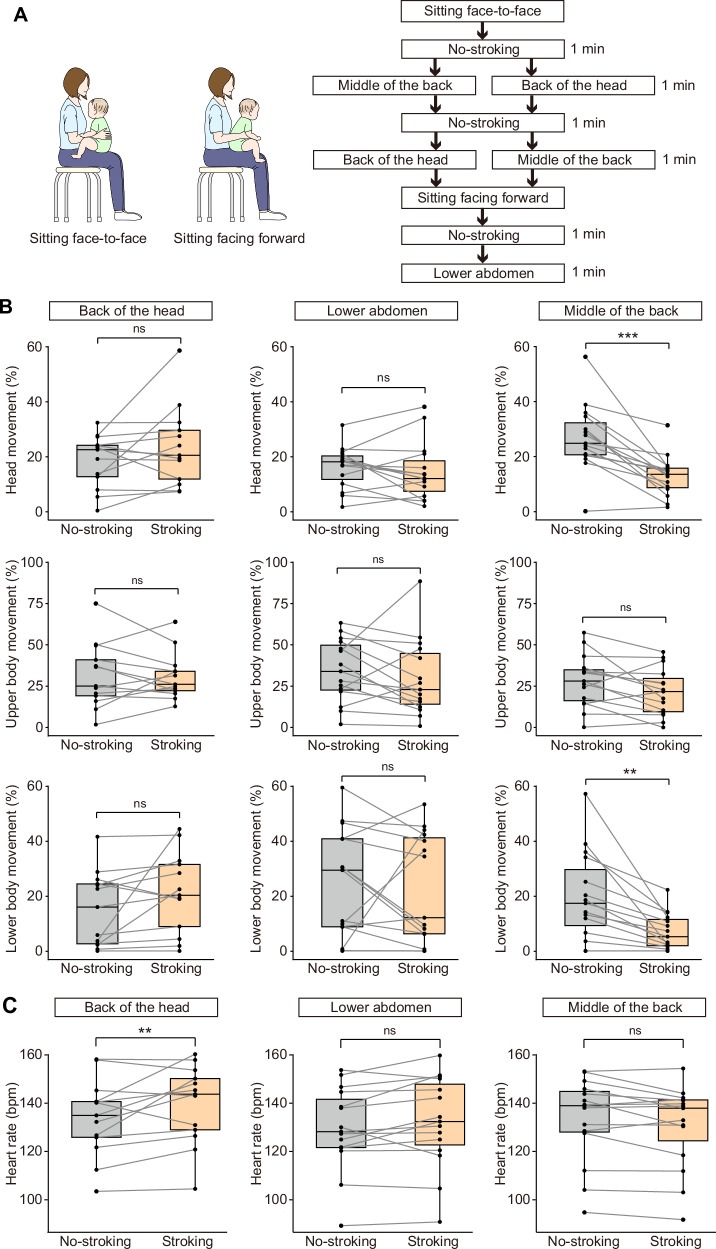
Calming responses to back stroking in human infants. **A** Illustration of two sitting positions used by mother–infant pairs (left) and experimental procedure when seated in a face-to-face position (right). **B** Comparison of spontaneous movements in the head, upper body, and lower body before and during stroking. The left panel shows data for back-of-head stroking, the middle for abdominal stroking, and right for back stroking. **C** Comparison of heart rate before and during stroking. The left panel shows data for back-of-head stroking, the middle for abdominal stroking, and right for back stroking. The boxes represent the 25th, median, and 75th percentiles, and the whiskers represent the lowest and highest data points within 1.5 times the interquartile range from the 25th and 75th percentiles. *n* = 15 (7 males and 8 females) in the lower abdomen and middle back stroking, and *n* = 13 (5 males and 8 females) in the back-of-head stroking. ^*^p < 0.05, ^**^p < 0.01. ns: not significant.

To characterize stroking parameters, we retrospectively quantified stroke velocity from video recordings in a subset of 13 mother–infant dyads. Mean stroking velocities (±SD) were 7.81 ± 2.24 cm/s for the back of the head, 12.40 ± 3.07 cm/s for the abdomen, and 10.53 ± 2.69 cm/s for the back, indicating that mothers typically stroked within or slightly above the velocity range reported to optimally engage C-tactile afferents in humans^19^. The sitting posture—whether the mother and infant faced each other or both faced forward—was left to the participants’ discretion. For mother–infant pairs who initially adopted a face-to-face position, the experiment began with stroking either the back or back-of-head (Fig. 1A). All analyses included only data from infants who did not cry during the procedures (7 males and 8 females for the back and lower abdomen stroking tasks; 5 males and 8 females for the back-of-head stroking task). To control for potential confounders such as trial order and individual variability, we analyzed the changes in spontaneous movements and heart rate using linear mixed-effects models (LMMs). Spontaneous movements and heart rate during the 1-min stroking period were compared with those during the 1-min pre-stroking period for each body part. Movements were categorized into three body regions: the head, upper body, and lower body. During the back-of-head stroking task, no significant changes in spontaneous movement were observed in any body region compared with the pre-stroking period (Fig. 1B, left, *p* > 0.05, see Supplementary Table 1). Similarly, stroking of the lower abdomen did not result in significant changes in movement (Fig. 1B, middle, *p* > 0.05, see Supplementary Table 1). In contrast, during back stroking, spontaneous movements in the head and lower body were significantly reduced compared to the pre-stroking period (Fig. 1B, right; Cohen’s *d* = 1.29, *p* < 0.001 for head movement; *d* = 0.83, *p* = 0.0015 for lower body movement, see Supplementary Table 1). No significant reduction was observed in upper body movement (*p* = 0.17). Within the subset of 13 mother–infant dyads with available stroke velocity data, we further tested whether individual differences in back stroking speed predicted the magnitude of movement reduction. Pearson correlations between back stroking speed and changes in movement during back stroking were not significant for either head movement (*r* = −0.06, *p* = 0.84) or lower body movement (*r* = −0.48, *p* = 0.098), suggesting that, within the observed range of velocities, the calming effect of back stroking on spontaneous movements did not strongly depend on stroke speed. No significant sex differences were found in movement change scores across all three body areas (LMM, *p* > 0.05 in head, upper body, and lower body movements).

Next, we compared the heart rate before and during each stroking task. The LMM analysis revealed a significant main effect of trial order (*p* = 0.028), indicating a gradual increase in baseline heart rate across the session, which was statistically controlled for. We also found a significant interaction between timing and body region (*p* = 0.028). Post-hoc comparisons showed that during back-of-head stroking, the heart rate significantly increased compared to the pre-stroking period (Fig. 1C, left; Supplementary Table 1, *d* = −0.67, *p* = 0.0096), suggesting an arousal response. Although excluded from the analysis, the only infant who cried during the mother–infant sessions did so during back-of-head stroking. No significant difference in heart rate was observed during abdominal stroking (Fig. 1C, middle; *d* = −0.35, *p* = 0.16). Back stroking induced a reduction in spontaneous movement but did not significantly decrease heart rate after controlling for order effects (Fig. 1C, right; *d* = 0.27, *p* = 0.28) and, unlike back-of-head stroking, was not associated with an arousal-related increase in heart rate. Furthermore, no significant changes were observed in the root mean square of successive differences (RMSSD), a representative index of heart rate variability (HRV), across any condition (LMM, *p* > 0.05, see Supplementary Table 1), indicating that the observed heart rate changes were not accompanied by robust changes in vagal-mediated HRV. There was no significant sex difference in heart rate across all three body areas. These findings indicate that the effects of maternal stroking vary depending on the body regions to be stroked. While stroking the back of the head induces arousal, back stroking effectively induces behavioral calming and may help prevent increases in physiological arousal.

### Mouse dams lick the back of their pups more often than their chests

**Fig. 2:**
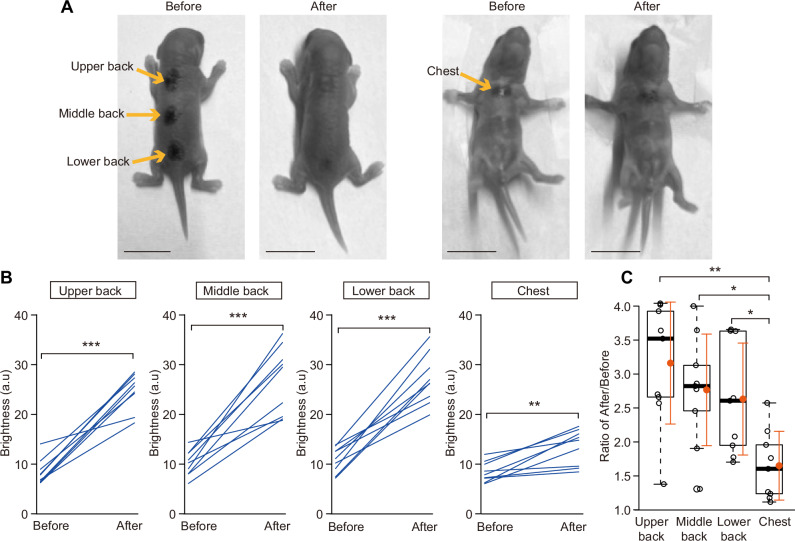
Region-specific quantification of maternal licking in neonatal mice. **A** Dorsal (left) and ventral (right) views of PND 2 mouse pups marked with ink. “Before” refers to the time point immediately after marking, prior to returning the pup to the home cage. “After” refers to the time point 4 h after the pup was returned to the home cage. **B** Within-individual changes in brightness for the upper back, middle back, lower back, and chest regions between before and after the 4 h of home cage exposure. **C** Comparison of brightness changes across different body regions. The boxes represent the 25th, median, and 75th percentiles, and the whiskers represent the lowest and highest data points within 1.5 times the interquartile range from the 25th and 75th percentiles. *n* = 9 (5 males and 4 females). Scale bars, 1 cm. ^*^p < 0.05, ^**^p < 0.01, ^***^p < 0.001.

It is well known that many quadrupedal mammalian parents, including rats and mice, frequently engage in licking behavior toward their offspring. This parental licking delivers tactile stimulation to the pups’ skin that resembles the sensory input induced by stroking. To investigate whether the reduction in spontaneous movement observed in human infants in response to back stroking also occurs in mouse pups, we first examined the preference of mouse dams for licking either the dorsal or ventral surfaces of their pups. Postnatal day (PND) 2 pups were removed from the home cage and marked on both the dorsal and ventral sides using a water-sensitive black ink that is removable through maternal licking. The brightness of each marked area was quantified both immediately before and 4 h after the pups were returned to the home cage with their dams (Fig. 2A). The results showed that brightness significantly increased on the upper back, middle back, lower back, and chest, indicating that these areas had been licked (Fig. 2B, Paired *t*-test with Holm correction, upper back: *t* = −9.67, df =  8, *p* = 1.088e-05, middle back: *t* = −7.045, df = 8, *p* = 0.00011, lower back: *t* = −8.69, df = 8, *p* = 2.41e-05, chest: *t* = −4.60, df = 8, *p* = 0.0018). When comparing dorsal and ventral areas, all three dorsal areas exhibited significantly greater increases in brightness, suggesting that dams licked the dorsal side more frequently with their moist tongues (Fig. 2C, One-way ANOVA, *F*[3, 17.23] = 8.47, *p* = 0.001, Pairwise comparison with Holm correction, *p* = 0.0046 in upper back vs. chest, *p* = 0.020 in middle back vs. chest, *p* = 0.037 in lower back vs. chest).

### Back stroking mimicking the maternal licking induces NREM sleep in mouse pups

**Fig. 3:**
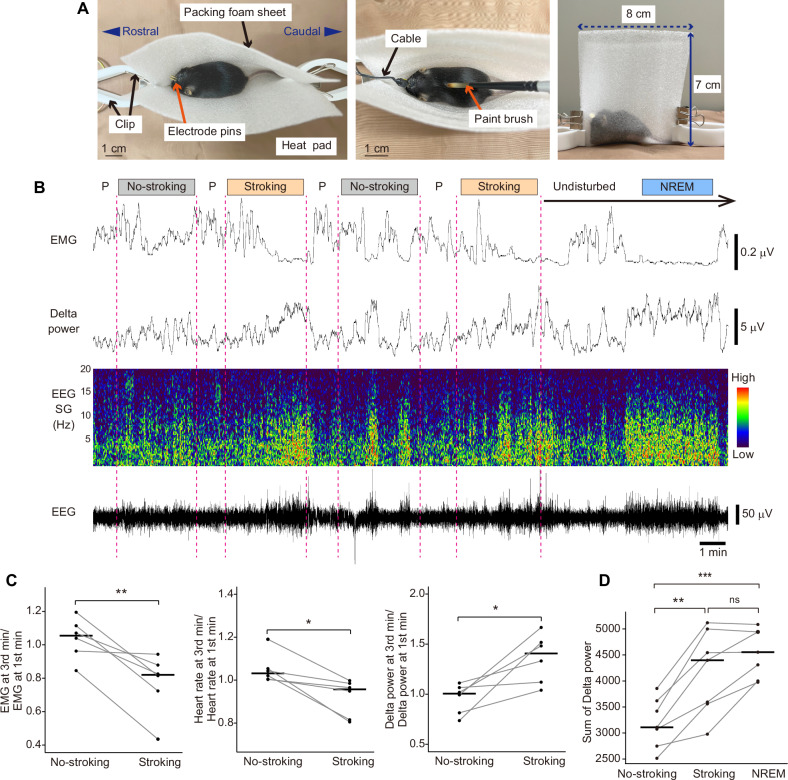
Calming responses to back stroking in mouse pups. **A** Three photographs of the experimental setup showing a gently restrained mouse pup undergoing simultaneous EEG, EMG, and ECG recordings during back stroking. **B** Representative traces showing changes across four conditions: pinna stroking (P), a no-stroking period (No-stroking), a back-stroking period (Stroking), and an undisturbed period. Panels display (from top to bottom): EMG, delta power, EEG spectrogram (SG), and raw EEG waveform. **C** Comparison of the first and last 1-min segments within 3-min no-stroking and stroking periods. Plots show ratio changes in EMG, heart rate, and delta power. **D** Comparison of total delta power during no-stroking, stroking, and NREM sleep periods. *n* = 6 (3 males and 3 females) in **C** and *n* = 7 (4 males and 3 females) in **D**. The horizontal line indicates the median. ^*^p < 0.05, ^**^p < 0.01, ^***^p < 0.001. ns: not significant.

As the findings demonstrated that mouse pups frequently receive maternal contact on the back, we next examined how back stroking affects their behavioral and physiological responses. To isolate the influence of back stroking, C57BL/6 (B6) pups were temporarily separated from their dam and gently restrained between soft packing foams. This setup allowed us to deliver controlled back stimulation with a soft brush that mimics the dam’s licking behavior, while electroencephalogram (EEG), ECG and electromyography (EMG) were recorded simultaneously (Fig. 3A). Calibration with a miniature pressure sensor showed that the average force (mean ± SD) was 0.059 ± 0.015 N for back stroking and 0.034 ± 0.012 N for pinna stroking, indicating that the brush was applied with a firm but consistent pressure across calibration trials (Supplementary Fig. 1). Based on video analysis, average stroking speeds were 8.43 ± 1.62 cm/s for the back and 7.24 ± 2.04 cm/s for the pinna, which fall within the velocity range typically used to activate C-fiber low-threshold mechanoreceptors, although we did not quantify the actual licking speed of mouse dams. A high-frequency band of EEG, such as 120–200 Hz or 110–300 Hz, contains components comparable to those of EMG, allowing for sleep-wake state identification^20,21^. Here, the 130-250 Hz signal extracted from the EEG of pups was used as the EMG signal (Supplementary Fig. 2). At PND14, pups implanted with EEG electrodes and ECG wires were placed in a narrow enclosure, and underwent a 3-min no-stroking period followed by a 3-min back-stroking period (Fig. 3B). Pups made little attempt to escape and instead exhibited intermittent movements, such as pressing their noses in the corners or shifting their body orientation (Supplementary Movie 1). To elevate the pups’ arousal levels before each period, an unpleasant tactile stimulus was applied by pinna stroking for 1 min. As a result, continuous back stroking led to a decrease in EMG activity and an increase in EEG delta power, resembling muscle tone and EEG patterns typically observed during non-rapid eye movement (NREM) sleep (Fig. 3B). Quantitative analysis revealed that continuous back stroking significantly decreased EMG activity and heart rate. Notably, interval estimation indicated that these physiological changes were substantial (Fig. 3C; paired *t*-test with Holm correction; EMG: *t* = 4.26, df = 5, *p* = 0.008; Heart rate: *t* = 3.52, df = 5, *p* = 0.017; Delta power: *t* = −3.46, df = 5, *p* = 0.018, see Supplementary Table 2). There was no significant sex difference in EMG, heart rate and delta power (Welch’s *t*-test, *p* > 0.05, Supplementary Table 3). Furthermore, comparisons of delta power across periods revealed that delta power during stroking was significantly higher than during no-stroking period and reached levels comparable to those observed during NREM sleep (Fig. 3D, Repeated-measures ANOVA, *F* [2, 12] = 43.03, *p* < 0.001, generalized η² (η²_g_) = 0.51, Pairwise comparison with Holm correction, Stroking vs. No-stroking (*p* = 0.003), NREM vs. No-stroking (*p* < 0.001)). The difference between Stroking vs. NREM was not statistically significant (*p* = 0.056), supporting the similarity in delta power between these states (see Supplementary Table 4). No significant sex differences were found in the sum of rectified EEG delta power (Welch’s *t*-test, *p* > 0.05, Supplementary Table 5). Thus, continuous back stroking in mouse pups, like in human infants, suppressed spontaneous movement and prevented physiological arousal. Additionally, EEG recordings indicated that back stroking effectively induced NREM sleep in mouse pups.

### Back stroking has a stress buffering effect on mouse pups

**Fig. 4:**
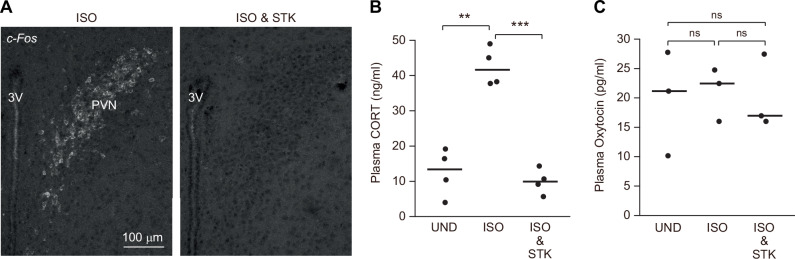
Back stroking acts as a stress-buffering stimulus in mouse pups. **A** Comparison of *c-Fos* mRNA expression in the paraventricular nucleus (PVN) of the hypothalamus between isolated pups (ISO) and pups stroked during isolation (ISO & STK). **B** Plasma corticosterone (CORT) levels in pups under undisturbed conditions (UND), isolation, or stroking during isolation. **C** Plasma oxytocin levels in pups under undisturbed conditions, isolation, or stroking during isolation. In **B**, *n* = 4 per group (2 males and 2 females). In **C**, *n* = 3 per group (2 males and 1 female). 3V, third ventricle; PVN, paraventricular nucleus. The horizontal line indicates the median. ^**^p < 0.01, ^***^p < 0.001. ns: not significant.

Affiliative physical contact between parent and offspring plays a crucial role in the development of stress resilience^22^. To examine whether back stroking confers stress-buffering effects, we conducted experiments using mouse pups. On PND14, pups were randomly assigned to one of three groups: (1) an isolation group, in which pups were placed alone in a narrow enclosure for 30 min (isolation stress); (2) an isolation-plus-stroking group, in which pups were placed alone in the enclosure and continuously stroked along the back with a brush throughout the 30-min isolation period; and (3) an undisturbed control group, in which pups remained in the home cage with their dams and littermates without any experimental manipulation. Stress exposure is known to activate the hypothalamic–pituitary–adrenal (HPA) axis, eliciting endocrine responses via the activation of corticotropin-releasing hormone (CRH) neurons in the paraventricular nucleus (PVN) of the hypothalamus^23^. The isolation group showed substantially higher expression of *c-Fos* mRNA, a marker of neuronal activity, in the PVN compared with the isolation-plus-stroking group (Fig. 4A). Consistently, the isolation group exhibited significantly higher corticosterone (CORT) levels compared to the other two groups at the end of 30 min session (Fig. 4B). A one-way ANOVA showed a large effect of condition (F[2, 5.59] = 46.39, *p* = 0.00033, η²_g_ = 0.91, 95% CI [0.76, 1.00]). Pairwise comparison with Holm correction indicated that CORT levels were higher in the isolation group than in the isolation-plus-stroking group (*p* = 0.00042) and the undisturbed group (*p* = 0.0011), whereas CORT levels in the isolation-plus-stroking group were comparable to those in the undisturbed group, with no significant difference (Fig. 4B; *p* = 0.54). No significant sex differences were found in each group (2-3 males, 2 females, Welch’s *t*-test, *p* > 0.05, Supplementary Table 6). Similar results were observed in pups at PND11, which corresponds to the late phase of the stress hyporesponsive period^24^, indicating that the stress-buffering effect of stroking emerges prior to the end of this period (Supplementary Fig. 3; One-way ANOVA, *F*[2, 7.82] = 145.6, *p* = 6.48 × 10⁻⁷, η²g = 0.97, 95% CI [0.92, 1.00]). As for oxytocin, a hormone involved in the formation of social bonds^25^, no significant differences in its concentration were observed among the three groups on PND14 (Fig. 4C; One-way ANOVA, *F*[2, 3.75] = 0.033, *p* = 0.97, η²_g_ = 0.01, 95% CI [0.00, 1.00]). Together, these findings demonstrate that affiliative tactile stimulation induced by back stroking has a stress-buffering effect in developing mice.

### Artificially reared pups fail to show drowsiness and stress buffering effect induced by the back stroking

During infancy, mammals exhibit various innate reflexes, such as the sucking reflex, in which pups begin to suck in response to tactile stimulation on the roof of the mouth. Fixed motor or postural responses to specific sensory stimuli are not limited to the very early stage of development. For example, Transport Response emerges from PND8 to PND16^26^. These findings prompted us to examine whether the calming effect of back stroking is innate or acquired through early-life tactile experiences, particularly those provided by maternal caregiving. To deprive pups of maternal licking and other parental contact, we employed artificially reared (AR) protocol with scheduled milk-feeding during the first 2 weeks after birth. We then compared their responses to back stroking with those of maternally reared (MR) pups by assessing changes in EMG, heart rate, EEG delta power and plasma CORT levels.

**Fig. 5:**
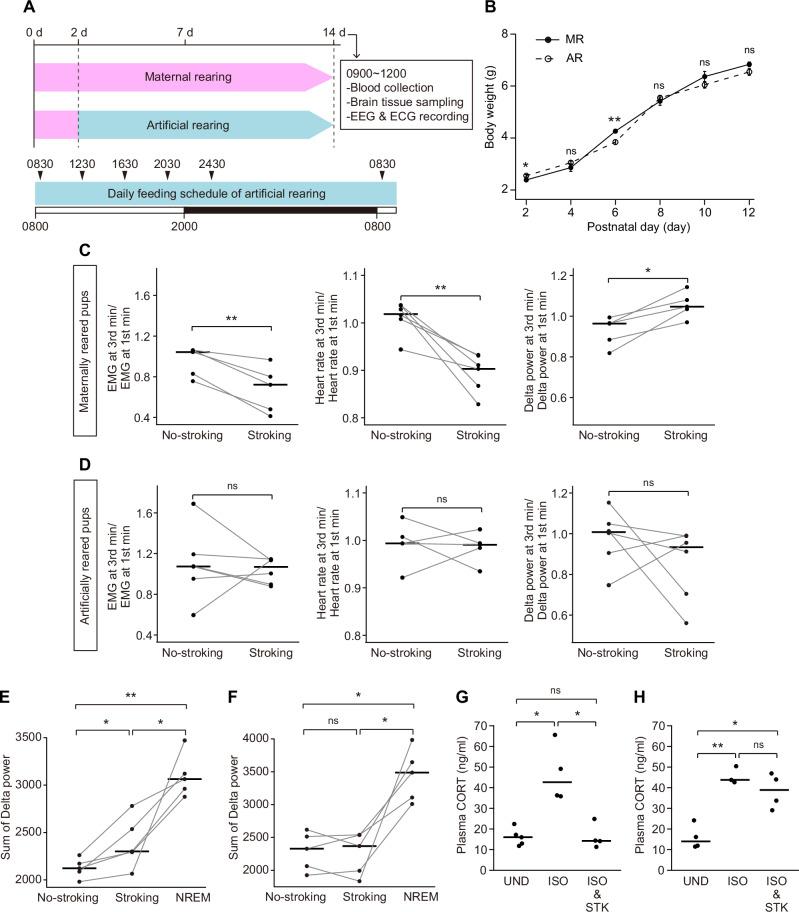
Stroking fails to induce sleep onset or stress buffering in artificially reared pups. **A** Experimental timeline for artificially reared pups, including scheduled feeding times and the sequence of behavioral and physiological assessments. White and black bars indicate the light and dark phases, respectively. **B** Comparison of body weight between maternally reared (MR) and artificially reared (AR) pups. **C**, **D** Comparison of the first and last 1-min segments within 3-min no-stroking and stroking periods. Plots show ratio changes in EMG, heart rate, and delta power in MR (**C**) and AR pups (**D**). In **C**, EMG and delta power, *n* = 5 (3 males and 2 females) due to EEG wire disconnection; heart rate, *n* = 6 (3 males and 3 females). In **D**, EMG and delta power, *n* = 6 (3 males and 3 females), except for heart rate, *n* = 5 (2 males and 3 females) due to ECG wire disconnection. Comparison of total delta power during no-stroking, stroking, and NREM sleep periods in MR (**E**) and AR (**F**) pups. *n* = 5 (2 males and 3 females) and *n* = 5 (3 males and 2 females) in **E** and **F**, respectively. Plasma CORT levels in MR (**G**) and AR (**H**) pups under undisturbed conditions (UND), isolation (ISO), or stroking during isolation (ISO & STK). *n* = 4–5 (2–3 males and 2 females) per group and n = 3–4 (1–2 males and 2 females) per group in **G** and **H**, respectively. Error bars represent mean ± SD in **B**. The horizontal line indicates the median. ^*^p < 0.05, ^**^p < 0.01. ns: not significant.

During the optimization of AR conditions, we initially used B6 mice or B6 × ICR hybrids. However, pups from these strains consistently failed to survive beyond the first postnatal week when reared artificially, indicating that the artificial rearing is particularly challenging in these backgrounds. We therefore selected the inbred ICR strain for all subsequent experiments due to its high survival rate, robust milk consumption, and consistent weight gain under artificial rearing conditions (Fig. 5A, and Supplementary Movie 2). Each feeding session included genital stimulation to induce urination and defecation, followed by weighing before and after milk ingestion. Milk intake was deemed complete when the pup spontaneously rejected the custom-designed artificial nipple. Throughout the rearing period, experimenters wore gloves, minimized handling, and refrained from grooming or stroking the pups. Our artificial rearing protocol supported overall weight gain comparable to that of the MR group (Fig. 5B; Welch’s *t*-test: PND2, *t* = 2.63, df = 12.70, *p* = 0.021; PND4, *t* = 1.19, df = 14.22, *p* = 0.26; PND6, *t* =  –3.78, df = 11.97, *p* = 0.0026; PND8, *t* = 0.72, df = 18.68, *p* = 0.48; PND10, *t* = –1.37, df = 18.96, *p* = 0.19; PND12, *t* = –1.85, df = 23.39, *p* = 0.077). AR pups, which are not licked by their dams, often have fur soiled with milk and feces and appear unkempt, but they exhibit no apparent motor abnormalities. Eyelid opening in AR pups occurred from PND12 to PND13, which was comparable to that in MR pups. There were no significant differences between MR and AR pups in the latency to perform the righting reflex at PND12 or in the duration of immobilization during the Transport Response at PND13, a filial-specific calming reaction observed during parent–infant transport in mammals^27^ (Supplementary Fig. 4A, B, righting reflex: Welch’s *t*-test: *t* = 0.38, df = 11.94, *p* = 0.71, Transport Response: Welch’s *t*-test: *t* = 0.15, df = 9.99, *p* = 0.88). A small number of pups were raised beyond weaning to assess potential behavioral abnormalities. At 8 weeks of age, no significant differences were observed between MR and AR mice in immobility time during a 6-min tail suspension test, a widely used behavioral assay for depressive-like behavior^28^ (Supplementary Fig. 4C, Welch’s *t*-test, *t* = –0.33, df = 5.59, *p* = 0.75, *n* = 4 per group).

On the morning of PND 13, we placed both AR and MR ICR pups in a narrow enclosure as performed with B6 pups and recorded EMG, heart rate and EEG during no-stroking and back-stroking periods. In MR pups, as observed in maternally reared B6 pups, back stroking significantly reduced EMG and heart rate and increased delta power compared to the preceding no-stroking periods. Interval estimation indicated that these changes were substantial (Fig. 5C; Paired *t*-test with Holm correction; EMG: *t* = 5.08, df = 4, *p* = 0.0071; Heart rate: *t* = 4.72, df = 5, *p* = 0.0052; Delta power: *t* = −4.55, df = 4, *p* = 0.010, see Supplementary Table 2). In contrast, AR pups showed no such reduction in EMG or heart rate, nor any increase in delta power upon back stroking, with 95% confidence intervals for the mean differences including zero (Fig. 5D; Paired *t*-test with Holm correction; EMG: *t* = 0.43, df = 5, p = 0.68; Heart rate: *t* = 0.30, df = 4, *p* = 0.78; Delta power: *t* = 1.13, df = 5, *p* = 0.31, see Supplementary Table 2). Although the delta power during stroking was smaller than that observed during NREM sleep in MR pups, it was significantly higher than during the no-stroking condition, indicating that stroking stimulation induces a drowsy state (Fig. 5E, A repeated-measures ANOVA, *F* [2, 8] = 24.27, *p* = 0.001, η²_g_ = 0.82, Pairwise comparison with Holm correction: Stroking vs. No-stroking, *p* = 0.040; NREM vs. No-stroking, *p* = 0.007; Stroking vs. NREM, *p* = 0.040; see Supplementary Table 4). In AR pups, delta power during both no-stroking and stroking periods was similar, and both were significantly lower than delta power during NREM sleep (Fig. 5F, A repeated-measures ANOVA, F [2, 8] = 22.25, *p* = 0.001, η²_g_ = 0.77, Pairwise comparison with Holm correction, Stroking vs. No-stroking, *p* = 0.71; NREM vs. No-stroking, *p* = 0.017; Stroking vs. NREM, *p* = 0.020, see Supplementary Table 4). In both AR and MR pups, no significant sex differences were found in EMG, heart rate, delta power, or the sum of delta power (Welch’s *t*-test, *p* > 0.05, Supplementary Tables 3 and 5).

We next examined whether 30 min of continuous back stroking could buffer isolation-induced stress in MR and AR pups. As observed in B6 pups, maternally reared ICR pups subjected to stroking during isolation showed CORT levels comparable to those of undisturbed controls, indicating effective stress buffering (Fig. 5G; One-way ANOVA, *F* [2, 5.28] = 8.049, *p* = 0.025, η²_g_ = 0.75, 95% CI [0.15, 1.00], Pairwise comparison with Holm correction, *p* = 0.0047 in isolation vs. isolation-plus-stroking, *p* = 0.0047 in isolation vs. undisturbed, *p* = 0.98 in undisturbed vs. isolation-plus-stroking). In contrast, AR pups showed no such reduction in CORT levels. Their CORT concentrations remained as elevated as those of AR pups subjected to isolation without stroking (Fig. 5H; One-way ANOVA, *F* [2, 5.28] = 27.69, *p* = 0.0016, η²_g_ = 0.83, 95% CI [0.54, 1.00], Pairwise comparison with Holm correction, *p* = 0.20 in isolation vs. isolation-plus-stroking, *p* = 0.0016 in isolation vs. undisturbed, *p* = 0.012 in undisturbed vs. isolation-plus-stroking). In both AR and MR pups, no significant sex differences were found in each group (Welch’s *t*-test, *p* > 0.05, Supplementary Table 6).

Taking together, these results suggest that early-life exposure to maternal care including physical contact contributes to the development of physio-behavioral responses to stroking. In the absence of such early maternal care, back stroking fails to induce drowsiness or attenuate stress responses in developing mice.

### *Cacna1b* knockdown pups fail to show drowsiness induced by the back stroking

**Fig. 6:**
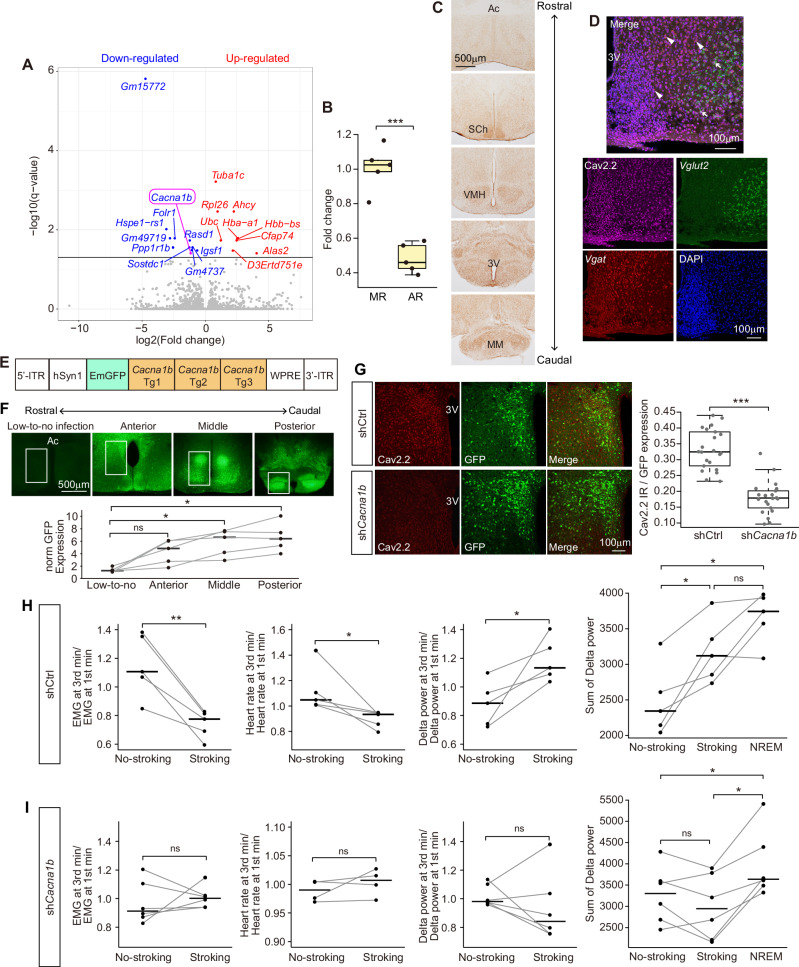
Hypothalamic *Cacna1b* expression regulates the stroking-induced calming response in mouse pups. **A** Volcano plot showing differentially expressed genes in the hypothalamus of maternally reared (MR) and artificially reared (AR) pups based on RNA-seq analysis. *n* = 4 (MR; 2 males and 2 females) and *n* = 3 (AR; 2 males and 1 female). **B** Quantitative PCR comparison of hypothalamic *Cacna1b* expression between MR and AR pups. n = 5 in each group (MR: 3 males and 2 females; AR: 3 males and 2 females). **C** Immunohistochemistry for Cav2.2, encoded by the *Cacna1b* gene, in the hypothalamus. **D** Combined immunohistochemistry and isHCR showing Cav2.2 protein, *Vglut2* mRNA, and *Vgat* mRNA in the hypothalamus. Arrows and arrowheads indicate Cav2.2/*Vglut2* and Cav2.2/*Vgat* double-positive neurons, respectively. **E** Schematic of the AAV vector carrying a construct for knockdown of the *Cacna1b* gene. **F** Expression and regional distribution of the *Cacna1b* knockdown vector in the hypothalamus visualized by GFP (top). Quantification of GFP expression in the non-infected area and in three infected hypothalamic regions (anterior, middle, and posterior parts) (bottom). Norm GFP indicates GFP expression normalized to fluorescence intensity measured in a non-infected cortical region. **G** GFP expression and Cav2.2 immunoreactivity (IR) in the hypothalamus of pups injected with shCtrl or sh*Cacna1b* vectors (left), and quantification of Cav2.2 immunoreactivity (right). *n* = 22 cells per group, prepared from two pups (one male and one female) in each rearing group (11 cells per pup). **H**, **I** Comparison of the first and last 1-min segments within 3-min no-stroking and stroking periods. Plots show ratio changes in EMG, heart rate, and delta power in pups with shCtrl (**H**) or sh*Cacna1b* (**I**). Total delta power during no-stroking, stroking, and NREM sleep conditions in pups with shCtrl (**H**) or sh*Cacna1b* (**I**). In **H**, EMG, heart rate, and delta power, *n* = 5 (2 males and 3 females). In **I**, EMG and delta power, *n* = 6 (3 males and 3 females), and heart rate *n* = 4 (2 males and 2 females). 3V, third ventricle; AC, anterior commissure; MM, mammillary nucleus; SCh, suprachiasmatic nucleus; VMH, ventromedial hypothalamic nucleus. The horizontal line indicates the median. ^*^p < 0.05, ^**^p < 0.01, ^***^p < 0.001. ns: not significant.

Next, we investigated differentially expressed genes between MR and AR pups in ICR strains. At PND13, total RNA was extracted from the hypothalamic region, given its pivotal role in sleep–wake regulation^29^ and stress responses^30^. Among the 55,414 genes analyzed, 20 genes were differentially expressed (FDR = 0.05) (Fig. 6A, Supplementary Fig. 5, Supplementary Table 7). Among these, we focused on the *Cacna1b* gene, which encodes the Cav2.2 voltage-gated calcium channel subunit, due to its essential role in synaptic neurotransmitter release in the central nervous system^31^ and its reported hyperactivation phenotype in knockout mice^32^.

Notably, *Cacna1b* mRNA exhibits a distinct temporal expression pattern during development, with elevated and widespread expression across the brain during the first two postnatal weeks, followed by a clear decline at the fourth week after weaning^33^.

RNA-seq analysis revealed a 45.14% reduction in *Cacna1b* mRNA expression in AR pups compared to MR pups (Fig. 6A, log_2_FC = −1.15, *q*-value = 3.93 × 10^−2^). Quantitative RT-PCR using total RNA from ICR pups confirmed this result, showing an approximately 48% reduction in *Cacna1b* mRNA expression in AR pups (Fig. 6B; Welch’s *t*-test: *t* = 7.54, df = 6.89, *p* < 0.001). Immunohistochemical analysis using PND14 B6 brain sections revealed broad Cav2.2 immunoreactivity throughout the hypothalamic region used for RNA extraction, spanning from the preoptic area to the retromammillary nucleus (Fig. 6C). Double staining demonstrated Cav2.2 expression in both glutamatergic and GABAergic neurons (Fig. 6D) as reported in previous studies^34^.

To examine the functional consequence of *Cacna1b* gene downregulation, we generated a short hairpin RNA (shRNA) plasmid construct (sh*Cacna1b*) containing three distinct target sequences (Fig. 6E, and Supplementary Table 8). B6 pups were bilaterally injected with the viral vector into the hypothalamus at PND1 and examined at PND14. At PND14, the present stereotaxic coordinates yielded widespread GFP expression throughout hypothalamic areas (Fig. 6F). We quantitatively assessed the knockdown efficiency of *Cacna1b* by analyzing immunohistochemical signal intensity in pups injected with either non-silencing control shRNA (shCtrl) or sh*Cacna1b*. In sh*Cacna1b* pups, Cav2.2 immunoreactivity, normalized to GFP expression, was reduced to nearly half, indicating that sh*Cacna1b* effectively downregulated Cav2.2 protein expression (Fig. 6G; Welch’s *t*-test: *t* = 8.83, df = 40.32, *p* < 0.001). We next examined whether back stroking in sh*Cacna1b* pups would induce reductions in spontaneous movement and heart rate, as well as an increase in delta power. In shCtrl pups, as observed in maternally reared B6 and ICR pups, continuous back stroking significantly reduced EMG activity and heart rate, while significantly increasing delta power compared to the preceding no-stroking period (Fig. 6H; paired *t*-test with Holm correction: EMG: *t* = 5.19, df = 4, *p* = 0.0065; heart rate: *t* = 2.81, df = 4, *p* = 0.048; Delta power: *t* = −3.23, df = 4, *p* = 0.032, Supplementary Table 2). Delta power during stroking was significantly higher than during the no-stroking period and reached levels comparable to those during NREM sleep (Fig. 6H; repeated-measures ANOVA: *F* [2, 8] = 18.94, *p* = 9.25e-04, η²g = 0.60; pairwise comparisons with Holm correction: stroking vs. no-stroking, *p* = 0.042; NREM vs. no-stroking, *p* = 0.11; stroking vs. NREM, *p* = 0.069, Supplementary Table 4).

In contrast, sh*Cacna1b* pups showed no reduction in EMG activity or heart rate, nor any increase in delta power following back stroking (Fig. 6I; paired *t*-test with Holm correction: EMG: *t* = −0.52, df = 5, *p* = 0.63; heart rate: *t* = −1.19, df = 3, *p* = 0.32; Delta power: *t* = 0.93, df = 5, *p* = 0.39, Supplementary Table 2). Furthermore, in sh*Cacna1b* pups, delta power during both no-stroking and stroking periods remained comparable and significantly lower than that observed during NREM sleep (Fig. 6I; repeated-measures ANOVA: *F* [2, 10] = 16.40, *p* = 6.96e-04, η²g = 0.27; pairwise comparisons with Holm correction: stroking vs. no-stroking, *p* = 0.18; NREM vs. no-stroking, *p* = 0.013; stroking vs. NREM, *p* = 0.013, Supplementary Table 4). These results indicate that somatosensory stimulation from maternal licking during early postnatal development contributes to the maintenance of Cav2.2 channel expression in the hypothalamus, and that Cav2.2-mediated neural signaling plays a critical role in the expression of NREM sleep induced by firm back stroking.

## Discussion

Here, we demonstrated that affiliative stroking stimulation rapidly induces a calming response characterized by reduced spontaneous movements in both human infants and mouse pups (Figs. 1 and 3). In this study, “calming” refers to a coordinated reduction in motor output and arousal-related physiological indices, not subjective affect or anxiety per se. In line with this operational definition, stroking additionally reduced heart rate, promoted sleep onset, and reduced stress levels in mice (Figs. 3 and 4). These effects were dependent on early-life maternal care involving close physical contact. Mouse pups that were artificially reared without maternal contact did not show calming or sleep-promoting responses to stroking. Instead, they exhibited elevated levels of stress markers (Fig. 5). At the molecular level, this developmental deficit was associated with reduced hypothalamic expression of *Cacna1b*, the gene that encodes the Cav2.2 voltage-gated calcium channel. Furthermore, targeted knockdown of *Cacna1b* in the hypothalamus of maternally reared pups eliminated the calming and sleep-inducing effects of stroking (Fig. 6). These results suggest the presence of a broadly shared mechanism by which affiliative tactile stimulation induces a calming response in mammalian young.

Neurological reflexes and behavioral responses that are fixed motor or behavioral reactions to specific sensory stimuli temporarily appear during infancy. These responses are essential for pup survival and facilitate parental caregiving behavior. For example, the sucking reflex, in which pups begin to suck in response to tactile stimulation on the roof of the mouth, enables pups to obtain milk from their mother’s nipples. Similarly, the Transport Response, a postural reaction elicited by pressure on the upper back, emerges between PND8 and PND16. This response helps parents safely carry their pups as the pups grow in size and weight^27^. In a similar developmental period, calming responses to back stroking are observed, which reduces pup activity and likely eases maternal labor by temporarily pacifying the pups. As weaning approaches, pups begin to behave more independently and forage for food in the home cage without relying on their dams. At this stage, back stroking no longer induces a calming effect and may even provoke agitation. Adult mice do not exhibit calming responses to back stroking by experimenters without prior training^35^, suggesting that this behavioral response is developmentally regulated. Although developmentally regulated reflexes and behavioral responses are generally innate and occur rapidly in response to sensory stimuli, the calming response to back stroking is distinct in that it takes minutes to develop and is acquired through parental care. Unlike rapid reflex arcs that involve direct pathways from sensory inputs to motor neurons, the calming effect may require sensory information to ascend to higher brain regions, where it modulates neural networks regulating sleep/wake state, particularly within the hypothalamus and other brain areas. Our current findings support the notion that the development of somatosensory-related reflexes and responses, such as the righting reflex and the Transport Response, can proceed largely independently of direct maternal contact in the early postnatal period. Previous studies have shown that fundamental aspects of somatosensory processing are established prenatally and continue to mature after birth^36^. Despite being deprived of maternal tactile interactions, artificially reared mice exhibited normal expression of the sucking reflex, righting reflex, and Transport Response. These mice also showed body weight growth comparable to those of maternally reared mice and no apparent abnormalities in sleep/wake behavior in the home cage. While we cannot rule out the possibility that artificial rearing disrupts several aspects of neural development, our data indicate that key sensory-motor responses are preserved under artificial rearing conditions.

In this study, infants who cried before or during stroking were excluded, which may have biased the sample toward relatively calm infants. In addition, because we prioritized having mothers reproduce their usual stroking, we did not use tools to standardize stroke speed or applied pressure. Future studies should test back stroking in infants who are crying or fussy and systematically examine variation in stroke speed and pressure. Such work will help define, in a developmentally informed manner, the stimulus features of stroking that are effective in real-world caregiving contexts.

Although gentle skin contact can evoke pleasant sensations via unmyelinated C-LTMRs^14^, and CT-targeted stroking in adults has been reported to reduce arousal and carry positive affective value^37^, our human and mouse studies used firmer pressure than is typically used in affective touch studies. Human caregivers were instructed to stroke the infant’s back with firm pressure to ensure that the stimulation was effectively delivered. In mouse pups, back stroking with a brush mimicked the pressure exerted by maternal licking and grooming. Sufficient pressure is required for dams to remove milk and excreta stains from the pup’s body, and in our artificial-rearing experiments, gentle touch or light brushing alone was insufficient to remove such stains from the body surface of artificially reared pups. Given that stroke velocities were within or slightly above the optimal range for C-LTMRs, these stimuli likely recruited both C-LTMRs and Aβ low-threshold mechanoreceptors. Because C-LTMRs can be activated across a range of forces, our data do not support the assertion that the applied forces were “too high” to engage C-LTMRs. Instead, we attribute the calming effects of back stroking to the combined activation of multiple low-threshold mechanoreceptor populations in both the dermis and subcutaneous tissue that converge onto central circuits regulating arousal. Beyond cutaneous afferents, subcutaneous connective tissues, including fascia, are also richly innervated and may contribute to the mechanosensory and autonomic consequences of stroking, which should be tested directly in future work.

Such painless, composite tactile pressure information is conveyed to the brain via spinal projection neurons (SPNs) in the dorsal horn. SPNs send direct and indirect projections to multiple brain regions, including broad areas of the hypothalamus^38^. The hypothalamus serves as a central integrative hub that regulates homeostasis and instinctive behaviors, including sleep/wakefulness and stress responses^29^. It is therefore likely that stroking the back modulates neuronal activity in the PVN of the hypothalamus either directly or indirectly, which may trigger a systemic calming response that integrates neuroendocrine, autonomic, and sleep–wake regulation in the offspring. Consistently, back stroking led to reduced heart rate and lowered circulating CORT levels, suggesting a shift toward parasympathetic dominance and decreased HPA axis activity. These physiological changes may, in turn, facilitate the induction of an NREMS-like state.

On the other hand, in human infants, stroking the head increased the heart rate. In mouse pups, stroking the pinna did not elicit a calming response. Stimulation of the back-of-head region, which is close to the face and neck, may therefore evoke a mild alerting or protective response without overt movement. Future studies incorporating additional state markers, such as pupil diameter or respiration, may help determine whether the heart-rate increase reflects attentional orienting rather than broader physiological arousal.

Consistent with the role of the hypothalamus in regulating sleep/wakefulness and stress responses, which are associated with the calming effects of back stroking^29^, artificially reared mice showed altered gene expression profiles in the hypothalamus at PND13. Artificially reared mice showed reduced expression of *Cacna1b*, which encodes the core subunit of the N-type voltage-gated calcium channel, Cav2.2, a channel known to mediate presynaptic neurotransmitter release. Furthermore, knockdown of *Cacna1b* in the hypothalamus of maternally reared mice abolished the calming response to back stroking. These findings suggest that maternal caregiving may help maintain sufficient levels of *Cacna1b* expression in the hypothalamus, thereby facilitating the postnatal development of the calming response to back stroking. At cerebellar and thalamic inhibitory synapses^39^, auditory brainstem excitatory synapses^40^, and the neuromuscular junction^41^, the responsiveness of Cav2.2 channels is rapidly lost between the first few postnatal days and the third postnatal week. These studies using rat pups suggest that Ca^2+^ channels involved in transmitter release undergo a developmental switch from N-type to P/Q-type at mammalian fast synapses^39–41^. It is therefore highly plausible that Ca^2+^ channels in the hypothalamus also undergo a developmental switch from N-type to P/Q-type during the first two postnatal weeks, and that this switching is impaired in artificially reared pups, which may partly account for their altered responses to back stroking compared with maternally reared pups. In adult mice, systemic *Cacna1b* deficiency results in a hyperactive phenotype and alterations in vigilance-state transitions^32,42^. Recent transcriptomic and chromatin accessibility studies in the preoptic area of the hypothalamus have revealed that sensory input shapes the postnatal maturation of neuronal subtypes, including the refinement of receptor expression patterns critical for cell-cell communication^43^. Relatedly, calcium-channel subtypes in peripheral low-threshold mechanosensory afferents have been implicated in social behaviors, including CaV3.2 signaling in C-LTMRs in adult mice^44^. Taken together, early-life physical contact with the dam likely plays a critical role in shaping synaptic transmission in the hypothalamus, in part through the regulation of specific calcium channel subtypes. This process may establish experience-dependent pathways through which affiliative stroking stimulation can promote sleep. However, since our genetic manipulation broadly affects the hypothalamus and *Cacna1b* is widely expressed throughout the hypothalamus, the specific neural populations or circuitries mediating this effect remain unidentified. The AR paradigm alters multiple variables beyond maternal physical contact, including maternal warmth/odor/social cues, nutrition, environmental conditions, and handling. Thus, the MR–AR comparison shows that maternal deprivation disrupts the calming response, but it does not isolate reduced tactile input as the sole causal factor. Handling-matched MR controls and an AR subgroup receiving standardized back stroking during rearing will help disentangle these confounds and clarify how early-life maternal caregiving shapes hypothalamic *Cacna1b* signaling.

In the present study, a single 30-min session of back stroking did not alter plasma oxytocin levels. Oxytocin has blood pressure-lowering effects and has been implicated in neural responses to social bonding and social touch^45–47^. In rats, either daily subcutaneous oxytocin administration or daily stroking for 5 min during the first few postnatal weeks leads to lower diastolic blood pressure in adulthood, and repeated early-life stroking has been proposed to enhance oxytocin levels^48^. In premenopausal women, higher self-reported frequency of hugs and massage with a spouse or partner has been associated with higher baseline plasma oxytocin levels^49^. Taken together, these findings raise the possibility that increases in peripheral oxytocin associated with stroking require repeated stimulation over multiple days. Therefore, the single-session stroking protocol used here may not have been sufficient to produce a detectable change in plasma oxytocin. In addition, peripheral oxytocin measures may not reflect central oxytocin release^50^. Our data support the possibility that stroking-induced calming relies on hypothalamic calcium-channel–dependent circuits, which may operate independently of oxytocin signaling.

The present study investigated the short-term effect of maternal deprivation during infancy and demonstrated that the calming effect induced by back stroking is a developmentally transient response. However, maternal deprivation may have long-term effects on social behavior, anxiety- and depression-like behavior, and innate behaviors such as parental care. This view is consistent with pioneering work by Seymour Levine, who showed that early handling and brief disruptions of the mother–infant relationship can produce enduring changes in endocrine stress responses and later behavior in animals^10,13^. In line with these findings, classic work in rats has shown that natural variation in maternal licking and grooming produces enduring changes in offspring stress reactivity and hippocampal glucocorticoid receptor (GR) expression, mediated in part by epigenetic modifications of the GR gene^12,51^. Although we did not examine epigenetic regulation in the present study, similar mechanisms may contribute to the altered stress responses and hypothalamic gene expression observed in AR pups. Given that repeated back-stroking-induced sleep may facilitate the maturation of sleep-wake patterns, it is possible that artificially reared mice may exhibit sleep and circadian abnormalities in adulthood. In humans, early-life experiences involving comforting physical contact with caregivers are known to shape both psychological well-being and physical health in later life. Individuals who have had limited exposure to positive and affectionate touch during early development, such as those who experienced neglect or abuse, often exhibit reduced sensitivity to the social and emotional aspects of affiliative tactile interaction in adulthood^52^. Although skin-to-skin contact and affiliative tactile stimulation between parents and infants are already practiced in clinical settings^53^, our findings provide a neurobiological basis for these interventions. Parental touch may help the maturation of hypothalamic neural systems and promote systemic calming, potentially supporting the development of stress resilience^37^. Future understanding of how peripheral tactile stimulation contributes to systemic calming through neural mechanisms may advance our understanding of pediatric conditions such as sensory hypersensitivity, hyposensitivity, and anxiety, and may inform the development of novel therapeutic approaches.

## Methods

### Human participants

Mother-infant pairs were recruited through advertisements distributed at regular events for postpartum parents at Toho University Omori Medical Center and at local childcare support facilities. All participants were Japanese. None of the participants had any serious physical or mental illnesses. All mothers were either full-time homemakers or on maternity leave at the time of the study. The mother’s mean age was 34 ± 4.34 years. Infants who cried before or during the experiments were excluded from the study. A total of 15 infants participated in each of the back and abdomen stroking tasks. Of the 15 infants initially recruited, two boys began to cry during the back-of-head stroking task, which was conducted after the abdomen and back stroking, and were therefore excluded. This resulted in a final sample of 13 infants for that task. The age and sex distribution of infants in the abdomen and back stroking tasks was 1.30 ± 1.43 years (7 males and 8 females). For the back-of-head stroking task, data from 13 infants (1.20 ± 1.15 years; 5 males and 8 females) were included in the final analysis. All procedures were approved by the Ethics Committee of the Faculty of Medicine at Toho University (Approval Protocol ID# A24085). Informed consent was obtained from all participants (or their parents/guardians). All ethical regulations relevant to human research participants were followed.

### Stroking experimental procedure in human infants

The studies were performed between 10:00 A.M. and 2:00 P.M. in the rectangular room (263 cm × 377 cm) at approximately 25 °C as described in our previous study^54^. When mothers and infants arrived at the university laboratory, the experimental procedure was explained to mothers, and they signed an informed consent form. We also obtained consent to use privacy-protected photos and videos by hiding participants’ faces. The mothers did not eat, drink or feed their infants 30 min before the experiment. The mothers did not use any fragrances or wristwatches. Before the experiment, the mothers changed clothes into plain shirts with short sleeves provided by the experimenters. To enhance the transmission of tactile stimulation from the mothers, the infants participated in this experiment wearing only diapers and baby underwear. Initially, to minimize stress for the infants, they could freely choose between sitting facing their mother on her lap or having both the mother and infant sitting facing forward (Fig. 1). If the infant started by sitting facing the mother, the task began with the mother stroking the infant’s back or the back of the head for 1 min. This is followed by stroking the back of the infant’s head or back for another 1 min, and finally, the infant is repositioned to sit facing forward, where the mother strokes the abdomen for 1 min to complete the measurements. On the other hand, if the infant starts by sitting facing forward, the task begins with stroking the abdomen for 1 min, followed by repositioning the infant to sit facing the mother, where the task of stroking the back or the back of the head for 1 min is carried out. Before and after each of the three tasks of stroking different body parts, a 1-min interval was inserted where the mother and infant sat without any stroking. During the measurement, the mother did not rock the infant, make eye contact, or talk to them.

### Behavioral and heart rate measurements in human infants

Two observers (S.Y. and M.Y.) independently recorded infants’ behaviors, including vocalizations and movements of the head, upper body, and lower body during each task. Using a stopwatch, they measured the duration of movement for each body part and calculated its proportion relative to the 1-min task duration. Inter-observer agreement was assessed using Cohen’s kappa, showing statistically acceptable reliability (*k* = 0.84 for head movement, *k* = 0.92 for upper body movement, and *k* = 0.85 for lower body movement). Three disposable ECG electrode patches (Nihon Kohden, Japan) were attached to the infants’ chests, following the method described in our previous study^54^. ECG signals were continuously recorded throughout the experiment. All sessions were videotaped from the front using Handycam camcorders (Sony, Japan) and BIMUTAS-Video software (KISSEI COMTEC, Japan). ECG signals were sampled at 1000 Hz (Nihon Kohden). Time-domain parameters, including mean R-R interval (RRI) and mean instantaneous heart rate, were calculated using R version 4.4.2. These parameters were compared between the baseline period (1 min prior) and during each stroking task (back, back of the head, and abdomen).

### Mice

All animal procedures were conducted in accordance with the Guidelines for Animal Experiments of Toho University and were approved by the Institutional Animal Care and Use Committee of Toho University (Approval Protocol ID #24-557). Approval was granted for the specific experiments described in this study. We have complied with all relevant ethical regulations for animal use. Breeding pairs of C57BL/6 J (B6) or ICR mice were obtained from Japan SLC (Hamamatsu, Japan) and CLEA Japan (Tokyo, Japan). The official nomenclature for B6 mice is C57BL/6JmsSlc from Japan SLC and C57BL/6JJcl from CLEA Japan. Similarly, the official names for ICR mice are Slc:ICR (Japan SLC) and Jcl:ICR (CLEA Japan). Mice were raised in our breeding colony under controlled conditions (12-h light/dark cycle, lights on at 8:00 A.M., 23 ± 2 °C, 55 ± 5% humidity, and ad libitum access to water and food). Mouse deliveries were monitored twice daily, at approximately 10:00 and 18:00. The day on which newborn pups were first observed was designated as the day of birth (postnatal day (PND) 0). Male and female pups were used at PND1-14, as specified for each experiment. On PND 1–2, litters were culled to six pups for B6 mice and to 14 pups for ICR mice, with approximately equal numbers of males and females. Surplus pups were euthanized by decapitation in accordance with institutional guidelines. All experiments were performed from 8:30 to 12:30. In the present study, no significant differences were observed in physiological or behavioral measurements between male and female pups. Therefore, data from both sexes were combined for analysis (Supplementary Tables 3 and 5).

### Assessment of maternal licking behavior

On PND 2, B6 pups were gently removed from the home cage, and three marks were placed on their back and one on their chest using a special marker (ZEBRA, Japan). The pups were then returned to the home cage with their dam. The marker used could not be removed by rubbing with dry cloth or paper but could be erased with a moist cloth or paper, allowing for evaluation of the extent of maternal licking. Approximately 4 h later, the pups were again removed from the home cage, and the visibility of each mark was photographed alongside a ruler for quantification. To evaluate maternal licking patterns on the pup’s body, images were converted to grayscale, and mean brightness values within defined regions of interest were compared between pups immediately after marking and those after a 4-h period in the home cage. Brightness values were quantified using ImageJ software (version 1.50i, NIH).

### Preparation of narrow enclosure and stroking procedure

A soft packing foam sheet was placed on top of a heating pad, and both ends were clipped to create a narrow enclosure approximately 7 cm in height and 8 cm in width. A new foam-sheet enclosure was prepared for each individual mouse pup and was not reused. Since mouse pups typically remain in close physical contact with their littermates—a behavior known as huddling, soft pressure applied to both sides of the body may mimic this huddling behavior, helping pups stay within confined spaces^55^. B6 and ICR pups were placed inside this setup at PND14 and PND13, respectively. After a 1-min resting period, the experimenter gently stroked the pup’s pinnae for 1 min and then left the pup undisturbed in the narrow enclosure for 3 min (no-stroking condition). The pup’s pinnae were then stroked again for 1 min, followed by 3 min of continuous back stroking with a soft paintbrush (stroking condition). Since mouse dams are known to thoroughly lick their pups to remove milk residues and feces that are firmly adhered to the pups’ bodies, maternal licking likely transmits tactile pressure not only to the surface of the skin but also to deeper subcutaneous tissues. To emulate this, the experimenter applied brush strokes with sufficient pressure to visibly move the pup’s back skin. To quantify the mechanical properties of the brush stimulation, we attached a miniature pressure sensor chip (Touchence Co., Ltd, Tokyo, Japan) to the tip of the brush and recorded the force at 100-ms intervals while the experimenter stroked the back or ear of five pups. We recorded videos of an experimenter stroking the backs or pinnae of five pups with a scale in the frame and quantified the stroking speed.

### EEG/ECG electrode implantation surgery

Male and female pups were implanted with EEG/ECG electrodes under anesthesia with isoflurane (2% for induction and 1% for maintenance) at PND13 in B6 mice and at PND12 in ICR strain. All surgical procedures were performed between 17:30 and 18:30. Prior to surgery, pups were fed soft milk agar using a spatula (15 g milk powder in 0.4% agarose; MARUKAN, Japan). The same milk agar was then placed in the postoperative cage. This procedure prevented deterioration of physical condition by the following morning, allowing for successful EEG/ECG recordings. After surgery, the pups were transferred to a new cage without the dam; the cage was equipped with a heating pad and a soft milk-agar block for weaning support. EEG and ECG recordings were conducted the following morning between 08:30 and 11:30. To avoid maternal rejection or dislodging of the head-mounted electrodes—caused by excessive licking or neglect in response to the protruding electrode pins—the pups were not returned to the home cage with the dam. The EEG/ECG electrode consisted of a four-pin gold-plated metal header with a 1.27 mm pitch. Two of the pins were connected to miniature screw electrodes (screw thread diameter: 1 mm; shaft length: 0.5 mm) via flexible Teflon-coated stainless-steel wires and were used for EEG recordings. The remaining two pins were connected to flexible Teflon-coated stainless-steel wires (1 cm and 7 cm in length, respectively) for ECG recordings. EEG electrodes were implanted epidurally over the parietal area (0.9 mm anterior to lambda, 0.9 mm lateral to the midline) referenced to the cerebellum area. For ECG recordings, one wire was inserted into the temporal muscle, and the other was tunneled subcutaneously and wound around the intercostal muscles near the heart. Under isoflurane anesthesia, pups were placed on a heating pad, the scalp was incised, and the skull was exposed. Small burr holes were made for the EEG screw electrodes, and the ECG wires were positioned as described above. The electrode assembly was secured using a high-performance instant adhesive, the incision was closed, and pups were allowed to recover on a warming pad before returning to the home cage.

### EEG/ECG recording and analysis

On the morning following surgery, each pup was placed in a narrow enclosure, and EEG and ECG signals were recorded at a sampling rate of 500 Hz using the VitalRecorder system (Kissei Comtec, Matsumoto, Japan). The onset and offset of both the no-stroking and stroking periods were annotated based on EEG and ECG signals, as well as synchronized video data displayed via SleepSign software (Kissei Comtec, Matsumoto, Japan). Epochs showing slow-wave activity accompanied by behavioral quiescence were extracted as sleep-like states. EEG and ECG signals were exported from SleepSign in CSV format. The EEG data were subsequently imported into Spike2 software (Cambridge Electronic Design). Electromyography components were extracted using a 130–250 Hz bandpass filter. We confirmed that this 130–250 Hz signal extracted from EEG in mouse pups was nearly identical to that of actual EMG (Supplementary Fig. 2). Therefore, in this paper, this extracted signal is denoted as EMG. Delta components were extracted using a 1–4 Hz bandpass filter. Both EMG and EEG signals were rectified and visualized for annotating the onset and offset of each event. Epochs previously identified as sleep-like in SleepSign corresponded to NREM sleep time windows in Spike2, during which the EMG signal was nearly absent and delta power was elevated. ECG data were processed using Microsoft Excel and R (version 4.4.2) software to calculate R-R intervals (RRI), and average heart rates were determined for the no-stroking, stroking, and NREM periods. Data from pups with electrode dislodgement or excessive artifacts were excluded from the analysis.

### Measurement of plasma corticosterone and oxytocin

To examine plasma corticosterone and oxytocin levels, three experimental groups were established: (1) pups isolated for 30 min inside the foam sheet enclosure, (2) pups continuously stroked on the back during the 30-min isolation period in the enclosure, and (3) pups that remained in the home cage with their dams without disturbance. At the end of the experimental period, pups were euthanized by decapitation, and blood samples were collected. Blood was collected into 1.5 ml microtubes containing 5 μl of heparin (1 unit/μl). The samples were centrifuged at 2000 × *g* for 10 min at 4 °C, and the resulting plasma was transferred to new tubes. Plasma corticosterone and oxytocin levels were quantified using AssayMax Corticosterone ELISA kit EC3001-1 (ASSAYPRO, MO, USA) and Oxytocin Enzyme Immunoassay Kit K048 (ARBOR ASSAYS, MI, USA), respectively.

### Preparation of brain sections for histological analyses

Mouse pups were anesthetized with isoflurane (3% for induction and 3% for maintenance) until loss of reflexes, and then transcardially perfused with 4% paraformaldehyde (PFA) in phosphate-buffered saline (PBS) for 2–4 h after the start of the light phase. The brains were postfixed in 4% PFA at 4 °C overnight, followed by cryoprotection in 30% sucrose in PBS for 2 days, embedded in O.C.T. Compound (Sakura Finetek, Tokyo, Japan), and stored at −80 °C. The frozen brains excluding the olfactory bulbs were cryosectioned coronally at a thickness of 50 μm. The sections were stored in an antifreeze solution (0.05 M phosphate buffer, 30% glycerol, 30% ethylene glycol) at −25 °C until use. To confirm the reproducibility of the staining, sections from two or three pups from different litters were used for each staining combination.

### In situ hybridization chain reaction using short hairpin DNAs and immunohistochemistry

To detect *c-Fos, Vglut2*, and *Vgat* mRNA expression, gene-specific DNA hairpins were prepared, and in situ hybridization chain reaction (isHCR) was performed using short hairpins, as described in previous studies^56^. For *c-Fos* probes, 18 target sequences were selected from the full-length cDNA using a homology search by NCBI BLASTn (https://blast.ncbi.nlm.nih.gov) (Supplementary Table 9). For combined isHCR and immunohistochemistry (IHC), tissue sections were blocked with 0.8% Block Ace (Dainihon Seiyaku) in PBST, followed by overnight incubation at 4 °C with anti-Cav2.2 antibody (1:600; ACC-002, Alomone Labs) diluted in 0.4% Block Ace in PBST. After three washes with PBST, the sections were incubated for 1 h at room temperature with goat anti-rabbit IgG conjugated to Alexa Fluor 568 (ab175471) and Hoechst 33342 (1 µg/ml). Sections were then mounted on glass slides and coverslipped using antifade mounting medium (VECTASHIELD Vibrance). For brightfield IHC, we employed a biotin-conjugated anti-rabbit secondary antibody (1:1000), an avidin-biotin-peroxidase complex (ABC kit; Vector Laboratories, CA, USA), and DAB substrate solution (Nacalai Tesque, Kyoto, Japan), following our previously described protocol^57^. Brightness values were quantified using ImageJ software (version 1.50i, NIH).

### Artificially reared pups

Artificially reared groups were established using ICR strain, following previously published protocols^58^. It is important to note that while this protocol isolates pups from maternal tactile stimulation, it also inevitably alters other environmental factors such as nutrition source, thermal regulation, and social interaction compared to maternal rearing. To mitigate litter-specific effects, experimental data were collected using artificially reared and maternally reared pups derived from at least three different litters. In both the artificially reared and the maternally reared groups, litters were culled to 14 pups per litter on PND1. Pups in the artificial rearing group were housed in small plastic containers maintained at high temperature and humidity, with 6–7 pups per container. Each pup was marked on the back with a pen for identification. From 08:30 to 24:30, pups were fed every 4 h. Prior to each feeding, gentle stimulation of the anogenital region was performed to induce urination and defecation, followed by weighing. After feeding, the pups were weighed again and returned to the container. Although milk intake varied among individuals, feeding was terminated when pups voluntarily stopped suckling on the artificial nipple, indicating satiety. During feeding, AR pups were briefly handled by the experimenter (wearing gloves) only for the purpose of milk delivery. Contact was kept to a minimum. Handling was limited to brief restraint for positioning and did not involve repetitive tactile stimulation to the back. This brief, instrumental handling was non-grooming and qualitatively distinct from maternal licking and stroking. No stroking, brushing, or fur grooming was performed to avoid tactile input that would mimic maternal care. On PND13, 30 min after the first morning feeding, EEG recordings and plasma CORT measurements were conducted.

### RNA-Seq

At PND13, AR and MR pups were deeply anesthetized with isoflurane and underwent terminal transcardial perfusion with 20% RNAlater (Thermo Fisher, MA, USA) diluted in ice-cold PBS, after which the brains were removed. Coronal brain slices of 1-mm thickness were prepared using a brain slicer (Muromachi, Tokyo, Japan), immersed in fresh RNAlater, and stored at −80 °C until further processing. Slices four through seven contained the hypothalamic region, including the medial preoptic area and the retromammillary nucleus. Under a stereomicroscope, the hypothalamic area surrounding the third ventricle was dissected using a surgical scalpel along a square mold (approximately 2 mm × 3 mm) created with a bent 33G needle. Total RNA was extracted using the NucleoSpin RNA kit (Takara Bio, Shiga, Japan) from four MR and three AR pups, each derived from three different litters.

RNA quality was verified via spectrophotometric analysis, and next-generation RNA sequencing (RNA-Seq) was performed (Takara Bio). All R scripts used for preprocessing, normalization, dimensionality reduction, and differential expression analysis are publicly available at https://github.com/Makoto-Kashima/mouse20250709/blob/main/seurat_TCC_pipeline.R. The code includes pipelines for performing Seurat (version 5.3.0)^59^-based normalization and visualization, and conducting differentially expressed gene analysis using the TCC package (version 1.48.0)^60^.

### Quantitative RT-PCR

Total RNA, as described above, was used for cDNA synthesis using the SuperScript III First-Strand Synthesis System (Invitrogen, MA, USA). Real-time quantitative PCR was performed using SYBR Green qPCR Master Mix (Thermo Fisher Scientific) following the manufacturer’s protocol. The following primers were used: *Gapdh* forward, 5′-AACTTTGTCAAGCTCATTTCCTGGT-3′; *Gapdh* reverse, 5′-GGTTTCTTACTCCTTGGAGGCCATG-3′; *Cacna1b* forward, 5′-CTCACGTCTCTTGTGGTCTTG-3′; *Cacna1b* reverse, 5′-TCCAGATTTGGTCCCTGTTATG-3′. All reactions were run in duplicate on an Applied Biosystems 7500 Fast Real-Time PCR System. Relative gene expression was calculated using the comparative Ct method (ΔΔCt method). The expression level of *Cacna1b* gene was normalized to that of *Gapdh* gene, and fold changes were determined using the formula 2^–ΔΔCt^.

### AAV preparation

Three distinct target sequences for *Cacna1b*, as recommended by the Broad Institute (Supplementary Table 8), were inserted into the pAAV-hSyn1EmGFP-3x-miR-shRNA*(Cacna1b*)-WPRE-bGHpA vector to generate a short hairpin RNA (shRNA) plasmid construct (sh*Cacna1b*), which was packaged into adeno-associated virus 8 (AAV8) particles by VectorBuilder (Chicago, IL, USA) (proposal/vector ID: P240502-1004wqp; lot no.: 240705AAVN04). The titer administered to mice in this study was 1.31 × 10^13^ GC/ml. A nonsilencing scrambled sequence was used to generate a control shRNA vector (shCtrl) (Supplementary Table 8); the shCtrl transfer plasmid was provided by a co-author (K. M.). The full-length nucleotide sequences of the sh*Cacna1b* and shCtrl transfer plasmids are provided as Supplementary Data 2 in FASTA format.

### Stereotaxic surgery

The stereotaxic apparatus was adapted from Chen et al.^61^. On PND1, pups were placed on crushed ice for 3–4 min. Complete cryoanesthesia was confirmed by cessation of all movements and a change in skin color from pink to purple. Cryoanesthetized pups received bilateral viral injections using glass capillaries connected to a Nanoject II system (Drummond, PA, USA). Prior to injection, the skin surface on the pup’s head was slightly pricked with a 33-gauge needle. Approximately 300 nL of virus (50.6 nL per injection, administered six times) was slowly injected into each hemisphere (relative to lambda: anteroposterior +0.5 mm, mediolateral ±0.5 mm, dorsoventral −3 mm). The capillary was withdrawn slowly after waiting for 1 min post injection. Pups were then placed on a warming blanket until fully recovered. The pups were tattooed on a paw or foot using a tattooing device (Natsume, Tokyo, Japan) for individual identification and then returned to their home cage. All injections were conducted between 09:00 and 12:00.

### Statistics and reproducibility

The data were preprocessed and visualized with Microsoft Excel and R version 4.4.2. All statistical analyses were conducted in R. For the human infant data, we fitted a linear mixed-effects model (LMM) to account for repeated measurements within infants, including random intercepts for each infant. The model included Timing (before vs. during) and Body region, as well as their interaction, as fixed effects, and we included stroke order and infant characteristics recorded in the protocol (age in months, sex, and birth order) as covariates. Sitting posture was not included as a separate covariate in the LMMs because it was strongly coupled with the body region being stroked in this protocol, as stroking was necessarily limited to the lower abdomen when mother–infant dyads were seated facing forward, making it difficult to statistically dissociate posture from body-region effects. For the mouse data, sample sizes were determined based on previous studies using similar experimental paradigms rather than statistical power analysis. The individual mouse was considered the experimental unit. Mice were randomly assigned to experimental groups. Experimenters were blinded to group allocation during the experimental data analysis for the *Cacna1b* knockdown experiments; however, blinding was not possible for the artificial rearing experiments due to visible differences in coat cleanliness (i.e., soiled fur) between groups. We used one-way ANOVA, paired *t*-tests, repeated-measures ANOVA, and Welch’s *t*-tests as appropriate. Effect sizes for ANOVAs are reported as generalized eta squared (η²_g_). Effect sizes for pairwise comparisons are reported as Cohen’s d (d) for paired samples. Significance was set at *p* < 0.05. When multiple pairwise comparisons were carried out within a family of tests, *p*-values were adjusted using Holm’s method. For the main analyses, we report effect sizes (Cohen’s d or η²_g_) and/or 95% confidence intervals (CIs) for all comparisons.

### Reporting summary

Further information on research design is available in the Nature Portfolio Reporting Summary linked to this article.

## Supplementary information

Supplementary Information

Description of Additional Supplementary Files

Supplementary Data 1

Supplementary Data 2

Supplementary Movie 1

Supplementary Movie 2

Reporting Summary

## Data Availability

The RNA-seq data have been deposited in the Gene Expression Omnibus (GEO) under accession number GSE305688. The plasmids generated in this study have been deposited in Addgene (Watertown, MA, USA): pAAV-shRNA-Cacna1b (ID: 254157) and the nontargeting control plasmid (ID: 254187). Numerical source data for the graphs and charts presented in this study are provided in the Supplementary Data 1 file. Full-length nucleotide sequences of the shCacna1b and shCtrl transfer plasmids are provided in Supplementary Data 2 (FASTA-formatted text). All other data supporting this study are available from the corresponding authors upon reasonable request.
