# Supplementary material for: Early-life maternal care is required for the typical development of calming responses to back stroking
Source: Commun Biol. 2026 Apr 10;9:894. doi: 10.1038/s42003-026-10012-6 (PMC13332149; doi:10.1038/s42003-026-10012-6)
Supplement: Supplementary file 1 — Supplementary Information [file 42003_2026_10012_MOESM1_ESM.pdf]

Supplementary information

# Early-life maternal care is required for the typical development of calming responses to back stroking

Sachine Yoshida, Akiko Harauma, Toru Moriguchi, Yousuke Tsuneoka, Kimiya Narikiyo, Kazuya Miyanishi, Makoto Kashima, Makoto Wada, Yu Hayashi, Hiromasa Funato

Correspondence:

sachine.yoshida@med.toho-u.ac.jp

hiromasa.funato@med.toho-u.ac.jp

This PDF file includes:
Supplementary Figures 1 to 5
Supplementary Tables 1 to 9
Captions for Supplementary Movies 1 and 2

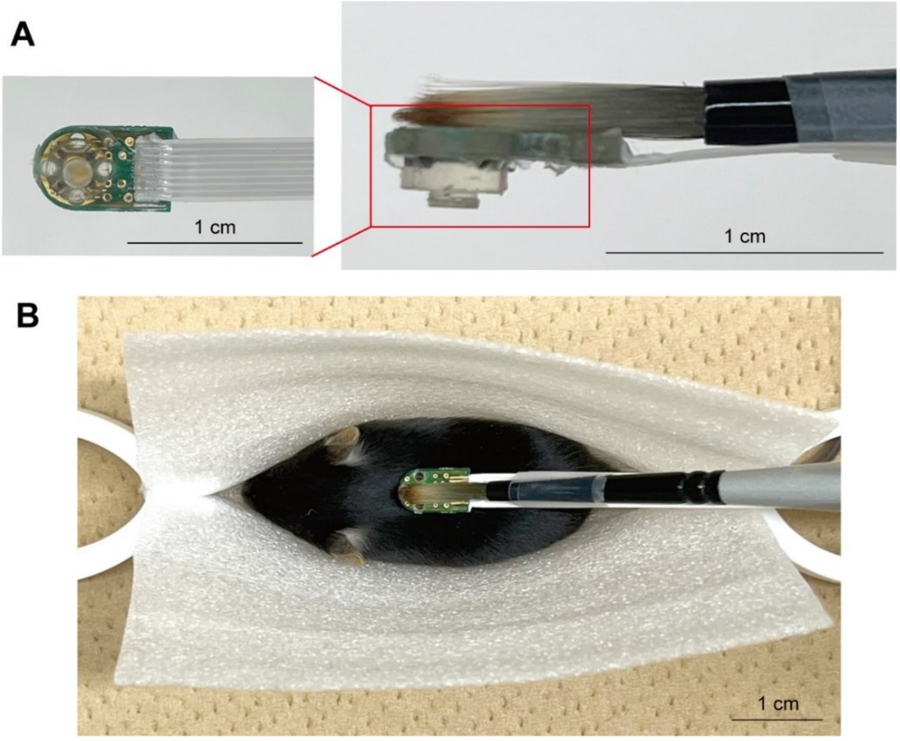


**Supplementary Figure 1. Calibration of contact force using a miniature pressure sensor**

(A) Side view of a brush equipped with a miniature pressure sensor. (B) Representative setup for measuring contact force during back stroking in a mouse pup.

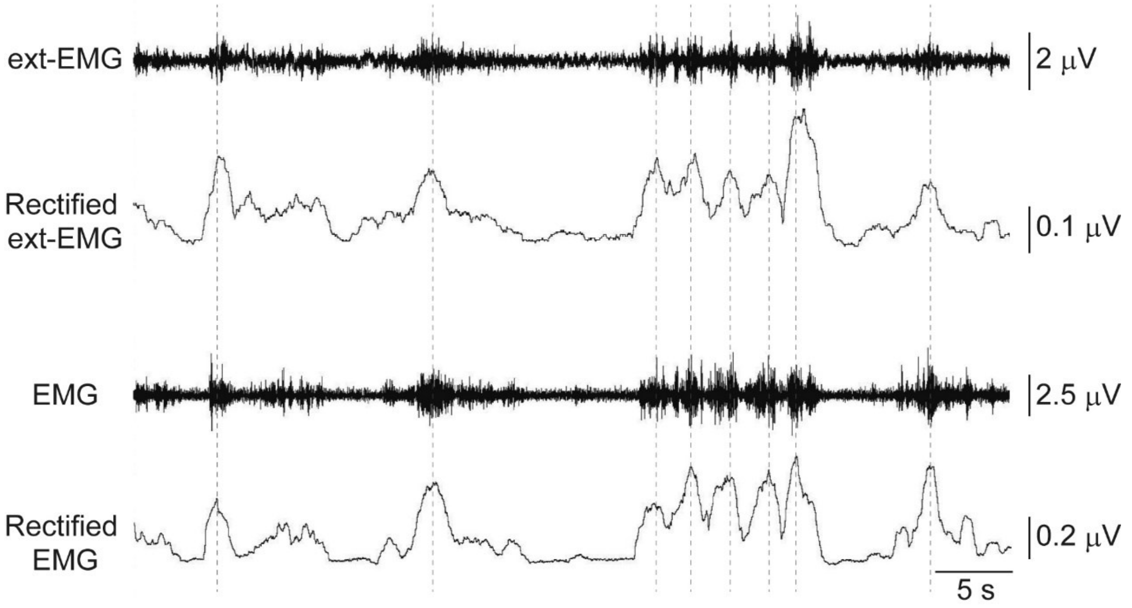


**Supplementary Figure 2. Comparison of EMG extracted from EEG and recorded neck EMG**

The top two panels show the EEG signal filtered in the 130–250 Hz range (ext-EMG, top) and its rectified signal (Rectified ext-EMG, second from the top). The bottom two panels show the raw EMG signal recorded from posterior neck muscles (EMG, second from the bottom) and its rectified signal (Rectified EMG, bottom).

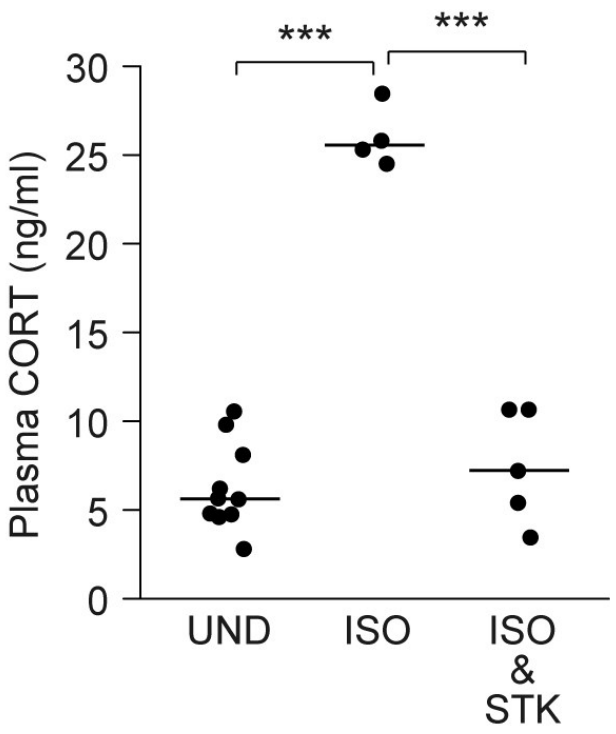


**Supplementary Figure 3. Plasma CORT level in PND11 pups.**

Comparison of plasma CORT levels among undisturbed, isolated, and stroked during isolation conditions at PND11. n = 10 (6 males and 4 females), 4 (2 males and 2 females) and 5 (3 males and 2 females) for UND, ISO and ISO & STK, respectively. The horizontal line indicates the median. ***: $p < 0.001$

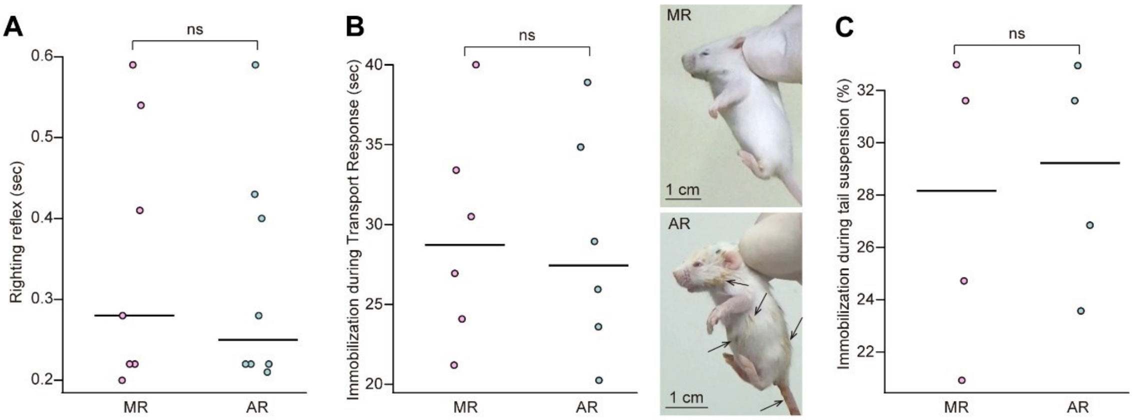


**Supplementary Figure 4. Comparison of whole-body movements between AR and MR mice.**

(A) The duration of righting reflex in MR and AR pups at PND12. (B) Immobilization time during Transport Response in MR and AR pups at PND13 (left) and their general appearance (right). Arrows indicate fecal or milk residues adhered to the fur surface. (C) Immobilization time during 6-min tail suspension test in MR and AR mice at 8 weeks. (A, B) n = 6 (3 males and 3 females). (C) n = 4 (2 males and 2 females). The horizontal line indicates the median.

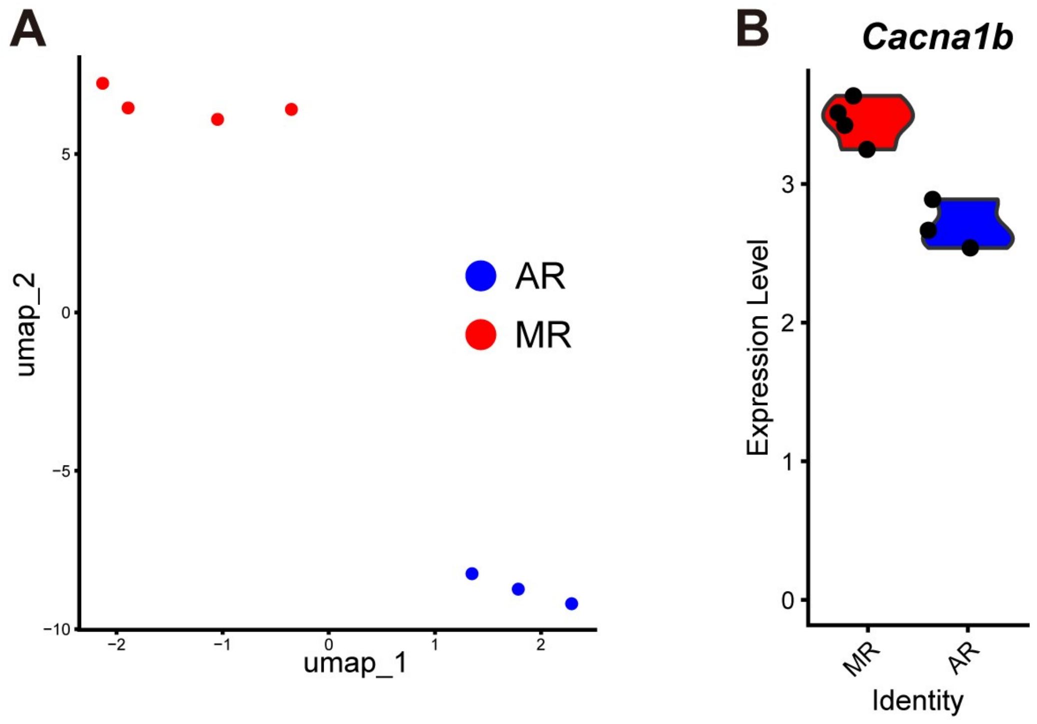


**Supplementary Figure 5. UMAP-based clustering reveals transcriptomic differences between MR and AR pups.**

(A) UMAP visualization of RNA-seq data reveals that MR and AR hypothalamic samples form distinct clusters, suggesting group-specific transcriptomic signatures. Each dot represents a sample (MR: 2 males and 2 females; AR: 2 males and 1 female). (B) The expression level of *Cacna1b* was lower in AR pups than in MR pups.

| Outcome Measure | Body Region | Mean Diff. (No-stroking – Stroking) | 95% CI for Mean Diff. [Lower, Upper] | Cohen's d | p-value |
|---|---|---|---|---|---|
| Head Movement | Head | -5.037 | [-10.81, 0.74] | -0.44 | 0.086 |
| | Abdomen | 1.71 | [-3.67, 7.079] | 0.16 | 0.53 |
| | Back | 13.87 | [8.50, 19.24] | 1.29 | < 0.001 |
| Upper Body Movement | Head | 2.61 | [-7.77, 12.99] | 0.13 | 0.62 |
| | Abdomen | 4.63 | [-5.032, 14.29] | 0.24 | 0.34 |
| | Back | 6.74 | [-2.92, 16.41] | 0.35 | 0.17 |
| Lower Body Movement | Head | -5.90 | [-14.17, 2.38] | -0.36 | 0.16 |
| | Abdomen | 1.72 | [-5.99, 9.42] | 0.11 | 0.66 |
| | Back | 12.78 | [5.068, 20.48] | 0.83 | 0.0015 |
| Heart Rate | Head | -5.69 | [-9.94, -1.44] | -0.67 | 0.0096 |
| | Abdomen | -2.79 | [-6.75, 1.17] | -0.35 | 0.16 |
| | Back | 2.15 | [-1.81, 6.11] | 0.27 | 0.28 |
| HRV (RMSSD) | Head | 1.64 | [-2.56, 5.84] | 0.19 | 0.44 |
| | Abdomen | 1.71 | [-2.20, 5.62] | 0.22 | 0.39 |
| | Back | 1.78 | [-2.13, 5.69] | 0.23 | 0.37 |

**Supplementary Table 1.** Statistical summary of behavioral and physiological responses to stroking across body regions in human infants.

| Mouse type (Related Figure) | Outcome | Mean Diff. (No-stroking – Stroking) | 95% CI for Mean Diff. [Lower, Upper] | t-value | df | p-value |
|---|---|---|---|---|---|---|
| MR B6 (Fig. 3C) | EMG | 0.27 | [0.11, 0.43] | 4.26 | 5 | 0.0080 |
| | Heart rate | 0.13 | [0.036, 0.23] | 3.52 | 5 | 0.017 |
| | Delta | -0.40 | [-0.70, -0.10] | -3.46 | 5 | 0.018 |
| MR ICR (Fig.5C) | EMG | 0.27 | [0.12, 0.422] | 5.08 | 4 | 0.0071 |
| | Heart rate | 0.12 | [0.053, 0.18] | 4.72 | 5 | 0.0052 |
| | Delta | -0.13 | [-0.21, -0.051] | -4.55 | 4 | 0.010 |
| AR ICR (Fig. 5D) | EMG | 0.063 | [-0.32, 0.44] | 0.43 | 5 | 0.68 |
| | Heart rate | 0.008 | [-0.063, 0.078] | 0.30 | 4 | 0.78 |
| | Delta | 0.13 | [-0.16, 0.41] | 1.13 | 5 | 0.31 |
| ShCtrl (Fig. 6H) | EMG | 0.41 | [0.19, 0.63] | 5.19 | 4 | 0.0065 |
| | Heart rate | 0.23 | [0.003, 0.45] | 2.81 | 4 | 0.048 |
| | Delta | -0.31 | [-0.57, -0.043] | -3.23 | 4 | 0.032 |
| Sh*Cacna1b* (Fig. 6I) | EMG | -0.037 | [-0.22, 0.15] | -0.52 | 5 | 0.63 |
| | Heart rate | -0.015 | [-0.055, 0.025] | -1.19 | 3 | 0.32 |
| | Delta | 0.087 | [-0.15, 0.33] | 0.93 | 5 | 0.39 |

**Supplementary Table 2.** Statistical comparisons of physiological indices during no-stroking and stroking periods in mice.

| Mouse type (Related Figure) | Outcome | Mean Diff. (Male-Female) | 95% CI for Mean Diff. [Lower, Upper] | t-value | df | p-value |
|---|---|---|---|---|---|---|
| MR B6 (Fig. 3C) | EMG | 0.11 | [-0.30, 0.51] | 0.81 | 3.18 | 0.47 |
| | Heart rate | 0.053 | [-1.75, 0.28] | 0.66 | 3.79 | 0.55 |
| | Delta | 0.060 | [-0.69, 0.81] | 0.23 | 3.62 | 0.83 |
| MR ICR (Fig.5C) | EMG | 0.096 | [-0.80, 0.99] | 0.72 | 1.39 | 0.57 |
| | Heart rate | 0.071 | [-0.051, 0.19] | 1.67 | 3.66 | 0.18 |
| | Delta | -0.077 | [-0.24, 0.089] | -1.98 | 2.020 | 0.19 |
| AR ICR (Fig. 5D) | EMG | 0.39 | [-0.37, 1.16] | 1.49 | 3.6 | 0.22 |
| | Heart rate | 0.05 | [-0.12, 0.20] | 1.01 | 2.67 | 0.39 |
| | Delta | 0.39 | [-0.073, 0.85] | 2.48 | 3.48 | 0.078 |
| ShCtrl (Fig. 6H) | EMG | 0.23 | [-0.215, 0.67] | 2.19 | 2.02 | 0.16 |
| | Heart rate | 0.30 | [-0.67, 1.27] | 3.04 | 1.13 | 0.18 |
| | Delta | 0.31 | [-1.60, 2.21] | 1.72 | 1.08 | 0.32 |
| Sh*Cacna1b* (Fig. 6I) | EMG | -0.11 | [-0.54, 0.32] | -0.72 | 3.67 | 0.51 |
| | Heart rate | -0.032 | [-0.24, 0.18] | -1.59 | 1.10 | 0.34 |
| | Delta | -0.106 | [-0.68, 0.46] | -0.53 | 3.83 | 0.63 |

**Supplementary Table 3.** Sex differences in stroking-induced changes in EMG, EEG delta power, and heart rate in mice

| Mouse type (Related Figure) | Comparison | Mean Diff. (A - B) | 95% CI for Mean Diff. [Lower, Upper] | t-value | df | p-value |
|---|---|---|---|---|---|---|
| MR B6 (Fig. 3D) | (A) Stroking vs (B) NREM | -373.8 | [-761.1, 13.6] | -2.36 | 6 | 0.056 |
| | (A) Stroking vs (B) No-stroking | 977.8 | [544.8, 1410.9] | 5.53 | 6 | 0.0030 |
| | (A) NREM vs (B) No-stroking | 1351.6 | [1088.7, 1614.5] | 12.6 | 6 | < 0.001 |
| MR ICR (Fig.5E) | (A) Stroking vs (B) NREM | -701.11 | [-1223.98, -184.24] | -3.76 | 4 | 0.040 |
| | (A) Stroking vs (B) No-stroking | 270.051 | [22.002, 518.10] | 3.02 | 4 | 0.040 |
| | (A) NREM vs (B) No-stroking | 974.16 | [586.45, 1361.87] | 6.98 | 4 | 0.0070 |
| AR ICR (Fig. 5F) | (A) Stroking vs (B) NREM | -1192.86 | [-1928.58, -457.15] | -4.50 | 4 | 0.020 |
| | (A) Stroking vs (B) No-stroking | -35.11 | [-280.94, 210.72] | -0.40 | 4 | 0.71 |
| | (A) NREM vs (B) No-stroking | 1157.76 | [561.19, 1754.32] | 5.39 | 4 | 0.017 |
| ShCtrl (Fig. 6H) | (A) Stroking vs (B) NREM | -477.8 | [-1016, 60.4] | -2.46 | 4 | 0.069 |
| | (A) Stroking vs (B) No-stroking | 698.9 | [171.7, 1226] | 3.68 | 4 | 0.042 |
| | (A) NREM vs (B) No-stroking | 1176.6 | [640.4, 1712.9] | 6.09 | 4 | 0.011 |
| Sh*Cacna1b* (Fig. 6I) | (A) Stroking vs (B) NREM | -991.4 | [-1507.3, -475.6] | -4.94 | 5 | 0.013 |
| | (A) Stroking vs (B) No-stroking | -278.2 | [-733.1, 176.7] | -1.57 | 5 | 0.18 |
| | (A) NREM vs (B) No-stroking | 713.2 | [314.4, 1112.1] | 4.60 | 5 | 0.013 |

**Supplementary Table 4.** Statistical summary of EEG delta power changes across different behavioral states in mice.

| Mouse type (Related Figure) | Outcome | Mean Diff. (Male-Female) | 95% CI for Mean Diff. [Lower, Upper] | t-value | df | p-value |
|---|---|---|---|---|---|---|
| MR B6 (Fig. 3D) | No-stroking | 60.70 | [-1074.3, 1195.7] | 0.15 | 3.96 | 0.89 |
| | Stroking | -478.1 | [-2762.9, 1806.7] | -0.68 | 2.89 | 0.55 |
| | NREM sleep | -138 | [-1250.6, 974.7] | -0.35 | 3.86 | 0.75 |
| MR ICR (Fig.5E) | No-stroking | 152.56 | [-61.78, 366.9] | 2.48 | 2.59 | 0.10 |
| | Stroking | 236.2 | [-1257.19, 1729.56] | 0.85 | 1.64 | 0.50 |
| | NREM sleep | -214.51 | [-782.75, 353.72] | -1.21 | 2.96 | 0.31 |
| AR ICR (Fig. 5F) | No-stroking | 156.50 | [-656.54, 969.54] | 0.62 | 2.95 | 0.58 |
| | Stroking | 115.10 | [-2357.94, 2588.14] | 0.30 | 1.44 | 0.80 |
| | NREM sleep | -481.76 | [-1726.43, 762.9] | -1.52 | 2.22 | 0.26 |
| ShCtrl (Fig. 6H) | No-stroking | -488.38 | [-1733.97, 757.2] | -1.34 | 2.68 | 0.28 |
| | Stroking | -429.48 | [-1541.84, 682.88] | -1.23 | 2.99 | 0.31 |
| | NREM sleep | -557.40 | [-2834.73, 1719.93] | -2.19 | 1.18 | 0.24 |
| Sh*Cacna1b* (Fig. 6I) | No-stroking | 146.40 | [-1591.77, 1884.57] | 0.24 | 3.76 | 0.82 |
| | Stroking | -468.44 | [-2499.19, 1562.32] | -0.72 | 3.14 | 0.52 |
| | NREM sleep | 409.46 | [-1788.68, 2607.6] | 0.59 | 3.02 | 0.60 |

**Supplementary Table 5.** Sex differences in EEG delta power during no-stroking, back stroking, and NREM sleep in mice.

| Mouse type (Related Figure) | Condition | t | df | p |
|---|---|---|---|---|
| MR B6 (Fig. 4B) | UND | -0.60 | 1.80 | 0.62 |
| | ISO | -0.34 | 1.76 | 0.77 |
| | ISO & STK | 1.99 | 2.00 | 0.19 |
| MR ICR (Fig.5G) | UND | 0.78 | 2.77 | 0.50 |
| | ISO | -0.52 | 1.39 | 0.67 |
| | ISO & STK | -1.19 | 1.16 | 0.42 |
| AR ICR (Fig.5H) | UND | 0.64 | 1.28 | 0.62 |
| | ISO | NA | NA | NA |
| | ISO & STK | -0.087 | 1.60 | 0.94 |

NA: No statistical test was performed for ISO AR pups (n = 3, 1 male and 2 females) due to limited sample size.

**Supplementary Table 6.** Statistical comparisons of plasma CORT levels between males and females.

| Gene Name | Gene Type | Transcript ID | Entrez Gene ID | Description |
|---|---|---|---|---|
| Gm15772 | processed pseudogene | ENSMUST00000118533.2 | - | predicted gene 15772 [Source:MGI Symbol;Acc:MGI:3805541] |
| Tuba1c | protein_coding | ENSMUST00000058914.10; ENSMUST00000230447.2 | 22146.0 | tubulin, alpha 1C [Source:MGI Symbol;Acc:MGI:1095409] |
| Rpl26 | protein_coding | ENSMUST00000073471.13; ENSMUST00000101014.9; ENSMUST00000128952.8; ENSMUST00000134403.2; ENSMUST00000138973.2; ENSMUST00000167436.3 | 19941.0 | ribosomal protein L26 [Source:MGI Symbol;Acc:MGI:106022] |
| Ahcy | protein_coding | ENSMUST00000054607.16; ENSMUST00000137242.2; ENSMUST00000146367.2 | 269378.0 | S-adenosylhomocysteine hydrolase [Source:MGI Symbol;Acc:MGI:87968] |
| Hspe1-rs1 | protein_coding | ENSMUST00000234633.2 | - | heat shock protein 1 (chaperonin 10), related sequence 1 [Source:MGI Symbol;Acc:MGI:1935159] |
| Gm49719 | lncRNA | ENSMUST00000231833.2 | - | predicted gene, 49719 [Source:MGI Symbol;Acc:MGI:6215192] |
| Hba-a1 | protein_coding | ENSMUST00000093209.4; ENSMUST00000142555.2 | 15122.0 | hemoglobin alpha, adult chain 1 [Source:MGI Symbol;Acc:MGI:96015] |

| | | | | |
|---|---|---|---|---|
| Folr1 | protein_coding | ENSMUST00000106981.8; ENSMUST00000106982.8; ENSMUST00000106983.8; ENSMUST00000106985.8; ENSMUST00000106986.9; ENSMUST00000123321.8; ENSMUST00000123630.8; ENSMUST00000124026.8; ENSMUST00000125298.2; ENSMUST00000126204.8; ENSMUST00000134145.8; ENSMUST00000140068.8; ENSMUST00000140584.2; ENSMUST00000150184.2; ENSMUST00000151706.8; ENSMUST00000155311.2 | 14275.0 | folate receptor 1 (adult) [Source:MGI Symbol;Acc:MGI:95568] |
| Hbb-bs | protein_coding | ENSMUST00000023934.8; ENSMUST00000131960.3; ENSMUST00000153218.2 | 100503605/15129 | hemoglobin, beta adult s chain [Source:MGI Symbol;Acc:MGI:5474852] |
| Cfap74 | protein_coding | ENSMUST00000050128.11; ENSMUST00000094408.10; ENSMUST00000105619.8; ENSMUST00000123952.9; | 544678.0 | cilia and flagella associated protein 74 [Source:MGI Symbol;Acc:MGI:1917130] |

| | | | | |
|---|---|---|---|---|
| | | ENSMUST00000129481.2; ENSMUST00000135407.8; ENSMUST00000144157.8; ENSMUST00000144625.2; ENSMUST00000151083.8; ENSMUST00000165947.3; ENSMUST00000238423.2; ENSMUST00000238620.2 | | |
| Rasd1 | protein_coding | ENSMUST00000062405.8 | 19416.0 | RAS, dexamethasone-induced 1 [Source:MGI Symbol;Acc:MGI:1270848] |
| Ubc | protein_coding | ENSMUST00000108707.3; ENSMUST00000136312.2; ENSMUST00000156249.2 | 22190.0 | ubiquitin C [Source:MGI Symbol;Acc:MGI:98889] |
| Gm4737 | protein_coding | ENSMUST00000059524.7 | 11615.0 | predicted gene 4737 [Source:MGI Symbol;Acc:MGI:3643647] |
| Ppp1r1b | protein_coding | ENSMUST00000078694.13; ENSMUST00000132443.8; ENSMUST00000133700.8; ENSMUST00000137634.2; ENSMUST00000147415.8; ENSMUST00000150762.8; ENSMUST00000152525.2 | 19049.0 | protein phosphatase 1, regulatory inhibitor subunit 1B [Source:MGI Symbol;Acc:MGI:94860] |

| | | | | |
|---|---|---|---|---|
| Sostdc1 | protein_coding | ENSMUST00000041407.7 | 66042.0 | sclerostin domain containing 1 [Source:MGI Symbol;Acc:MGI:1913292] |
| Matn2 | protein_coding | ENSMUST00000022947.7; ENSMUST00000163455.9; ENSMUST00000226766.2; ENSMUST00000227119.2; ENSMUST00000227759.2; ENSMUST00000228570.2 | 17181.0 | matrilin 2 [Source:MGI Symbol;Acc:MGI:109613] |
| Igsf1 | protein_coding | ENSMUST00000033442.14; ENSMUST00000072037.13; ENSMUST00000114891.2; ENSMUST00000114893.8; ENSMUST00000135492.2 | 209268.0 | immunoglobulin superfamily, member 1 [Source:MGI Symbol;Acc:MGI:2147913] |
| D3Ertd751e | protein_coding | ENSMUST00000026867.14; ENSMUST00000026868.13; ENSMUST00000108065.9; ENSMUST00000119572.8; ENSMUST00000120167.8; ENSMUST00000143841.8; ENSMUST00000146165.8; ENSMUST00000192193.6; ENSMUST00000192799.2; ENSMUST00000193075.6; | 73852.0 | DNA segment, Chr 3, ERATO Doi 751, expressed [Source:MGI Symbol;Acc:MGI:1289213] |

| | | | | |
|---|---|---|---|---|
| | | ENSMUST00000193228.6; ENSMUST00000194346.6; ENSMUST00000195030.2; ENSMUST00000195577.2; ENSMUST00000195882.6 | | |
| Cacna1b | protein_coding | ENSMUST00000041342.12; ENSMUST00000070864.14; ENSMUST00000100348.10; ENSMUST00000102939.9; ENSMUST00000114447.8; ENSMUST00000124183.2; ENSMUST00000125798.3; ENSMUST00000131861.2; ENSMUST00000133892.2; ENSMUST00000155356.4 | 12287.0 | calcium channel, voltage-dependent, N type, alpha 1B subunit [Source:MGI Symbol;Acc:MGI:88296] |
| Alas2 | protein_coding | ENSMUST00000066337.13; ENSMUST00000112715.2; ENSMUST00000134670.2; ENSMUST00000142474.2 | 11656.0 | aminolevulinic acid synthase 2, erythroid [Source:MGI Symbol;Acc:MGI:87990] |

**Supplementary Table 7.** Description of 20 differentially expressed genes.

| Target | Sequence |
| --- | --- |
| *Cacna1b* | CCTTACTTTCGGGATCTTT |
| | GCGCATCATACAATGACAT |
| | GCCGTAATATCATGGGATT |
| Nontargeting control | GCACTGGCGAGAGATGTAGTT |
| | GTGGTAATGTGCTTATTGTAT |
| | GCACAATACCGATAATCTGAT |

**Supplementary Table 8.** Three distinct target sequences for sh*Cacna1b* and shCtrl.

| Probe name | First probe | Second probe |
|---|---|---|
| cFos-1S10 | CGTCGGATGAAATGGTCGAAGTTTGGGGAAAGCCCG | TAGTCGGCGTTGAAACCCGAGAACAAAAGCCCATTAGAT |
| cFos-2S10 | CGTCGGATGAAGGGGAATGGTAGTAGGAAAGGCTGT | GAGCCCATGCTGGAGAAGGAGTCGGAAAGCCCATTAGAT |
| cFos-3S10 | CGTCGGATGAATGCGCAAAAGTCCTGTGTGTTGACA | AAAGTTGGCACTAGAGACGGACAGAAAAGCCCATTAGAT |
| cFos-4S10 | CGTCGGATGAAATGCTCTGCGCTCTGCCTCCTGACA | AGCTGCTCTACTTTGCCCCTTCTGCAAAGCCCATTAGAT |
| cFos-5S10 | CGTCGGATGAATCCGTTTCTCTTCCTCTTCAGGAGA | CCATCTTATTCCGTTCCCTTCGGATAAAGCCCATTAGAT |
| cFos-6S10 | CGTCGGATGAAGCAGACTTCTCATCTTCAAGTTGAT | AGCAGATTGGCAATCTCAGTCTGCAAAAGCCCATTAGAT |
| cFos-7S10 | CGTCGGATGAATCGGTGGGCTGCCAAAATAAACTCC | AAGGTCATCGGGGATCTTGCAGGCAAAAGCCCATTAGAT |
| cFos-8S10 | CGTCGGATGAAGCTTGGGCTCAGGGTCGTTGAGAAG | TGATGCTCTTGACTGGCTCCAAGGAAAAGCCCATTAGAT |
| cFos-9S10 | CGTCGGATGAAAAGGGTTCTGCCTTCAGCTCCACGT | GATGATGCCGGAAACAAGAAGTCATAAAGCCCATTAGAT |
| cFos-10S10 | CGTCGGATGAAATCTGGCACAGAGCGGGAGGTCTCT | TGCATAGAAGGAACCGGACAGGTCCAAAGCCCATTAGAT |
| cFos-11S10 | CGTCGGATGAACTCGGGCAGTGGCACGTCTGGATGC | GTGTTTCTCCTCTCTGTAATGCACCAAAGCCCATTAGAT |
| cFos-12S10 | CGTCGGATGAAAGGTCGACGGGAACCTTCGAGGGAA | GTTTCACGAACAGGTAAGGTCCTCCAAAGCCCATTAGAT |
| cFos-13S10 | CGTCGGATGAAGCAAGTCCTTGAGGCCCACAGCCTG | AGGACTGGAGGCCAGATGTGGATGCAAAGCCCATTAGAT |
| cFos-14S10 | CGTCGGATGAATCACTAGGAACAACACACTCCATGC | GCTCTACTAACTACCAGCTCTCAGGAAAGCCCATTAGAT |
| cFos-15S10 | CGTCGGATGAAGGTTAATTCCAATAATGAACCCAAC | AGCTGCACTAGATACAATCCAGCACAAAGCCCATTAGAT |
| cFos-16S10 | CGTCGGATGAACGCTATTGCCAGGAACACAGTAGGT | TAATATTGGTCGTTTCTAATTGGAAAAAGCCCATTAGAT |
| cFos-17S10 | CGTCGGATGAATGACGCTGAAGGACTACAGTACATG | CATGATCAGTAACATGACAATGAACAAAGCCCATTAGAT |
| cFos-18S10 | CGTCGGATGAAGAACATTCAGACCACCTCGACAATG | CGTTTTCATGGAAAACTGTTAATGTAAAGCCCATTAGAT |

**Supplementary Table 9.** isHCR probe sequences for mouse *c-Fos* gene

**Supplementary Movie 1.** Comparison of pups' responses to no stroking and back stroking.

**Supplementary Movie 2.** Milk feeding to artificially reared pups using a specialized nipple.
