# Supplementary material for: Early-life maternal care is required for the typical development of calming responses to back stroking
Source: Commun Biol. 2026 Apr 10;9:894. doi: 10.1038/s42003-026-10012-6 (PMC13332149; doi:10.1038/s42003-026-10012-6)
Supplement: Supplementary file 2 — Description of Additional Supplementary Files [file 42003_2026_10012_MOESM2_ESM.docx]

**Description of Additional Supplementary File**

File name: Supplementary data 1
Description: Numerical source data underlying the graphs and charts in the main figures (Excel).

File name: Supplementary data 2
Description: Full-length nucleotide sequences of the shCacna1b and shCtrl (nontargeting) transfer plasmids (text file; FASTA-formatted).

File name: Supplementary Movie 1
Description: Supplementary movie related to Fig. 3 (movie file).

File name: Supplementary Movie 2
Description: Supplementary movie related to Fig. 5 (movie file).
