# Supplementary material for: Early-life maternal care is required for the typical development of calming responses to back stroking
Source: Commun Biol. 2026 Apr 10;9:894. doi: 10.1038/s42003-026-10012-6 (PMC13332149; doi:10.1038/s42003-026-10012-6)
Supplement: Supplementary file 7 — Reporting Summary [file 42003_2026_10012_MOESM7_ESM.pdf]

nature portfolio

Corresponding author(s): Sachine Yoshida, Hiromasa Funato

Last updated by author(s): Mar 12, 2026

Reporting Summary

Nature Portfolio wishes to improve the reproducibility of the work that we publish. This form provides structure for consistency and transparency in reporting. For further information on Nature Portfolio policies, see our Editorial Policies and the Editorial Policy Checklist.

Statistics

For all statistical analyses, confirm that the following items are present in the figure legend, table legend, main text, or Methods section.

n/a	Confirmed	
☐	☒	The exact sample size (*n*) for each experimental group/condition, given as a discrete number and unit of measurement
☐	☒	A statement on whether measurements were taken from distinct samples or whether the same sample was measured repeatedly
☐	☒	The statistical test(s) used AND whether they are one- or two-sided<br>*Only common tests should be described solely by name; describe more complex techniques in the Methods section.*
☐	☒	A description of all covariates tested
☐	☒	A description of any assumptions or corrections, such as tests of normality and adjustment for multiple comparisons
☐	☒	A full description of the statistical parameters including central tendency (e.g. means) or other basic estimates (e.g. regression coefficient) AND variation (e.g. standard deviation) or associated estimates of uncertainty (e.g. confidence intervals)
☐	☒	For null hypothesis testing, the test statistic (e.g. F, t, r) with confidence intervals, effect sizes, degrees of freedom and P value noted<br>*Give P values as exact values whenever suitable.*
☒	☐	For Bayesian analysis, information on the choice of priors and Markov chain Monte Carlo settings
☒	☐	For hierarchical and complex designs, identification of the appropriate level for tests and full reporting of outcomes
☐	☒	Estimates of effect sizes (e.g. Cohen's *d*, Pearson's *r*), indicating how they were calculated

Our web collection on statistics for biologists contains articles on many of the points above.

Software and code

Policy information about availability of computer code

Data collection	1. Infant behavioral data were recorded using BIMUTAS-Video (KISSEI COMTEC, Japan).<br>2. EEG/ECG signals were acquired and annotated using VitalRecorder and SleepSign (KISSEI COMTEC, Japan), and analyzed in Spike2 (version 10.07, Cambridge Electronic Design).<br>3. Maternal licking behavior and immunohistochemistry images were quantified using ImageJ (version 1.50i, NIH).
Data analysis	1.Statistical analyses were conducted in R (version 4.4.2) using standard functions for a linear mixed-effects model, one-way ANOVA, repeated-measures ANOVA, Welch's t-test, paired t-test, and Holm's method for multiple-comparison correction.<br>2. Data preprocessing and visualization were performed using Microsoft Excel and R (version 4.4.2) with the tidyverse package.<br>3. RNA-seq data analysis was performed in R using the Seurat package (version 5.3.0) and the TCC package (version 1.48.0).<br>4. The full analysis pipeline is available at: https://github.com/Makoto-Kashima/mouse20250709/blob/main/seurat_TCC_pipeline.R

For manuscripts utilizing custom algorithms or software that are central to the research but not yet described in published literature, software must be made available to editors and reviewers. We strongly encourage code deposition in a community repository (e.g. GitHub). See the Nature Portfolio guidelines for submitting code & software for further information.

Data

Policy information about availability of data

All manuscripts must include a data availability statement. This statement should provide the following information, where applicable:

- Accession codes, unique identifiers, or web links for publicly available datasets
- A description of any restrictions on data availability
- For clinical datasets or third party data, please ensure that the statement adheres to our policy

The RNA-seq data generated in this study have been deposited in the Gene Expression Omnibus (GEO) under accession number GSE305688. All other data supporting the findings of this study are available within the paper and its supplementary information.

Research involving human participants, their data, or biological material

Policy information about studies with human participants or human data. See also policy information about sex, gender (identity/presentation), and sexual orientation and race, ethnicity and racism.

Reporting on sex and gender	Information on biological sex was collected from the infants' caregivers. Both male and female infants were included (abdomen/back: 7 males, 8 females; back-of-head: 5 males, 8 females). As the participants were infants, only biological sex was considered. Sex differences were analyzed, and no significant differences were found in any of the stroking tasks. All mothers who participated in the study were biological females who had given birth; no information on gender identity was collected.
Reporting on race, ethnicity, or other socially relevant groupings	All participants were Japanese. No other racial, ethnic, or socially relevant groupings were recorded.
Population characteristics	The study involved mother–infant pairs. Mothers were biological females, either full-time homemakers or on maternity leave, with a mean age of 34 ± 4.34 years. Infants had a mean age of 1.30 ± 1.43 years for the abdomen/back stroking tasks (7 males, 8 females) and 1.20 ± 1.15 years for the back-of-head task (5 males, 8 females). Infants who cried during the experiments were excluded. None of the participants had serious physical or mental illnesses.
Recruitment	Participants were recruited through advertisements distributed at postpartum events at Toho University Omori Medical Center and at local childcare support facilities.
Ethics oversight	All procedures were approved by the Ethics Committee of the Faculty of Medicine at Toho University (Approval Protocol ID# A24085).

Note that full information on the approval of the study protocol must also be provided in the manuscript.

Field-specific reporting

Please select the one below that is the best fit for your research. If you are not sure, read the appropriate sections before making your selection.

☒ Life sciences ☐ Behavioural & social sciences ☐ Ecological, evolutionary & environmental sciences

For a reference copy of the document with all sections, see nature.com/documents/nr-reporting-summary-flat.pdf

Life sciences study design

All studies must disclose on these points even when the disclosure is negative.

Sample size	Sample sizes were not predetermined by statistical power calculations. Instead, they were chosen based on prior publications in the field and our own pilot experiments using similar methods. For all main experiments, sufficient biological replicates (n) were included to allow reproducibility and to detect statistically significant differences with standard statistical analyses. The reproducibility of the observed effects across independent experiments supports the adequacy of the sample sizes used in this study.
Data exclusions	In the human infant experiments, infants who cried before or during the tasks were excluded from analysis. For animal experiments, no additional data were excluded.
Replication	All key experiments were repeated 2–3 times, and results were consistent across replications.
Randomization	The experiments were not randomized. Infants and mouse pups were assigned to experimental groups by balancing for sex and age.
Blinding	Investigators were blinded to group allocation until after data collection and analysis were completed.

Reporting for specific materials, systems and methods

We require information from authors about some types of materials, experimental systems and methods used in many studies. Here, indicate whether each material, system or method listed is relevant to your study. If you are not sure if a list item applies to your research, read the appropriate section before selecting a response.

Materials & experimental systems

n/a	Involved in the study
☐	☒ Antibodies
☒	☐ Eukaryotic cell lines
☒	☐ Palaeontology and archaeology
☐	☒ Animals and other organisms
☒	☐ Clinical data
☒	☐ Dual use research of concern
☒	☐ Plants

Methods

n/a	Involved in the study
☒	☐ ChIP-seq
☒	☐ Flow cytometry
☒	☐ MRI-based neuroimaging

Antibodies

Antibodies used	We used the following antibodies in this study: anti-Cav2.2 rabbit polyclonal antibody (1:600; ACC-002, Alomone Labs), goat anti-rabbit IgG (H+L) conjugated to Alexa Fluor 568 (1:1000; ab175471, Abcam), and biotin-conjugated anti-rabbit IgG (1:1000; BA-1000, Vector Laboratories).
Validation	The specificity of the anti-Cav2.2 antibody (ACC-002, Alomone Labs) was confirmed by the manufacturer (Western blot) and validated for immunohistochemistry (Tu et al., Am J Physiol Cell Physiol, 2014).

Animals and other research organisms

Policy information about studies involving animals; ARRIVE guidelines recommended for reporting animal research, and Sex and Gender in Research

Laboratory animals	C57BL/6J and ICR mice were obtained from Japan SLC and CLEA Japan.
Wild animals	Not applicable. The study did not involve wild animals.
Reporting on sex	Both male and female mouse pups were used in this study. Licking behavior was measured only in female dams.
Field-collected samples	Not applicable. The study did not involve samples collected from the field.
Ethics oversight	All animal procedures were conducted in accordance with the Guidelines for Animal Experiments of Toho University and were approved by the Institutional Animal Care and Use Committee of Toho University (Approval Protocol ID #24-557).

Note that full information on the approval of the study protocol must also be provided in the manuscript.

Plants

Seed stocks	N/A
Novel plant genotypes	N/A
Authentication	N/A
